# Food‐grade phytosome vesicles for nanoencapsulation of labile *C*‐glucosylated xanthones and dihydrochalcones present in a plant extract matrix—Effect of process conditions and stability assessment

**DOI:** 10.1002/fsn3.3730

**Published:** 2023-10-06

**Authors:** Chantelle Human, Marique Aucamp, Dalene de Beer, Marieta van der Rijst, Elizabeth Joubert

**Affiliations:** ^1^ Plant Bioactives Group, Post‐Harvest and Agro‐Processing Technologies Agricultural Research Council (Infruitec‐Nietvoorbij) Stellenbosch South Africa; ^2^ School of Pharmacy University of the Western Cape Bellville South Africa; ^3^ Department of Food Science Stellenbosch University Matieland (Stellenbosch) South Africa; ^4^ Biometry Unit Agricultural Research Council Stellenbosch South Africa

**Keywords:** bioactive phenolic compounds, food grade, functional food ingredient, nano‐phytosomes, powder stability

## Abstract

Phytosomes consist of a phytochemical bound to the hydrophilic choline head of a phospholipid. Their use in food products is gaining interest. However, literature on the use of food‐grade solvents, crude plant extracts as opposed to pure compounds, and unrefined phospholipids to prepare phytosomes is limited. Furthermore, studies on compound stability are lacking. This study aimed to develop nano‐phytosome vesicles prepared from inexpensive food‐grade ingredients to improve the stability of polyphenolic compounds. *Cyclopia subternata* extract (CSE) was selected as a source of phenolic compounds. It contains substantial quantities of *C*‐glucosyl xanthones, benzophenones, and dihydrochalcones, compounds largely neglected to date. The effect of process conditions on the complexation of CSE polyphenols with minimally refined food‐grade fat‐free soybean lecithin (PC) was studied. The PC:CSE ratio, sonication time, and reaction temperature were varied. This resulted in phytosomes ranging in vesicle size (113.7–312.7 nm), polydispersity index (0.31–0.48), and zeta potential (−55.0 to −38.9 mV). Variation was also observed in the yield (93.5%–96.0%), encapsulation efficiency (3.7%–79.0%), and loading capacity (LC, 1.3%–14.7%). Vesicle size and LC could be tailored by adjusting the sonication time and PC:CSE ratio, respectively. Chemical interaction between the lipid and the phenolic compounds was confirmed with nuclear magnetic resonance. Phytosomal formulation protected the compounds against degradation when freeze‐dried samples were stored at 25 and 40°C for 6 months at low relative humidity. The study provided valuable information on the importance of specific process parameters in producing food‐grade phytosomes with improved phenolic stability.

## Graphical abstract

Nano‐phytosome vesicles were prepared from inexpensive food‐grade ingredients for improved stability of xanthones, benzophenones, and dihydrochalcones present in an ethanolic extract of *Cyclopia subternata* (CSE). The effect of process conditions on the complexation of CSE with fat‐free soybean lecithin (40% phosphatidylcholine, PC) was studied.

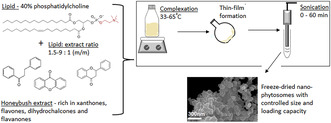

## 1 INTRODUCTION

Polyphenols are increasingly being added to a variety of food and beverage products to enhance bioactivity and/or functionality. Their use presents some challenges, as they have poor stability and bioavailability. One strategy for overcoming these obstacles is the use of nanocarriers (Nejatian et al., 2022; Zhang et al., 2022), such as nano‐phytosomes. Although nano‐phytosome technology has been developed for enhanced drug delivery, it offers an attractive option as a delivery vehicle for polyphenols with high hydrogen donor or acceptor capacity (Barani et al., 2021; Lu et al., 2019).

Phytosomes can also be used to produce food‐grade delivery systems (Ghanbarzadeh et al., 2016), however, studies on their application in food products are still limited. Similarly, studies on compound stability are also limited. Compound stability before ingestion is important to ensure a standardized dose of bioactive compounds is delivered, especially in food applications where processing for microbial safety is required. Recent examples of application in food products are nano‐phytosomes containing rutin (Babazadeh et al., 2017), resveratrol (Rabbani et al., 2021), and *Echinacea* extract (Molaveisi et al., 2021). In terms of stability of phytosomal formulations, good stabilization of rutin was achieved under pasteurization conditions (Babazadeh et al., 2017), vitexin was stable under both pasteurization and sterilization conditions (Peanparkdee & Yooying, 2023), and pomegranate peel extract showed improved stability of the polyphenols during storage (Dundar et al., 2023).

Contrary to liposomes where the phospholipid only surrounds the phenolic compounds, phytosomes are formed through hydrogen bonding between the polar heads of the phospholipid molecules and the phenolic compound in a molar‐specific ratio (Ghanbarzadeh et al., 2016). This is performed in a reaction medium that promotes the self‐assembly of the phospholipid into a vesicular structure (Kumar et al., 2020). In this form, the phytosome not only mimics the cell membrane but the phenolic compound is also stabilized by the hydrogen bond, resulting in increased stability and bioaccessibility (Ghanbarzadeh et al., 2016). However, the extent to which phytosomes outperform unformulated phenolic compounds is dependent on a variety of nano‐phytosome properties such as the vesicle shape and size, loading capacity (LC), and encapsulation efficiency (EE). These properties can be manipulated by varying the processing parameters such as the reaction time, temperature, and phospholipid‐to‐phenolic compound ratio, among others (Babazadeh et al., 2017; Saoji et al., 2016).

Typically, phytosomes are produced using phosphatidylserine, phosphatidylethanolamine, and phosphatidylcholine, pure bioactive phenolic compounds of interest, and aprotic solvents such as acetone, methylene chloride, and ethyl acetate (Ghanbarzadeh et al., 2016). However, if phytosomes are to be used in food products, the phospholipids, excipients, and solvents must be “generally recognised as safe” (GRAS) (Babazadeh et al., 2019). In addition, aqueous reaction media, unrefined phospholipid mixtures, and plant extracts instead of pure phospholipids and isolated phenolic compounds should be used to limit cost (Yokota et al., 2012). The use of such ingredients will also have a lesser impact on the environment (Cortes‐Clerget et al., 2021). Phytosomes prepared from such unrefined ingredients could likely result in more formulation challenges.

For the present study, we selected a bioactive extract of *Cyclopia subternata* (CSE) to produce a food‐grade phytosome. The extract was shown to enhance immune responses and induce Treg differentiation in mice at a human‐equivalent dose relevant to a cup of tea (Yoshida et al., 2020). Not only does *C. subternata* contain a unique spectrum of *C*‐glucosylated xanthones, benzophenones, and dihydrochalcones but these compounds have also been shown to degrade during high‐temperature processing in a model ready‐to‐drink beverage (Human et al., 2021). Although the phenolic compounds exhibited high stability during a spray‐drying process (Pauck et al., 2017), storage of the spray‐dried extract also resulted in phenolic losses (De Beer et al., 2018). Subsequently, the preparation of nano‐phytosome vesicles (CSE‐NV) loaded with *C. subternata* extract and fat‐free soybean lecithin (PC) containing 40% phosphatidylcholine were prepared. The impact of various process parameters (PC:CSE ratio, sonication time, and reaction temperature) on the properties linked to phytosome formation efficiency (vesicle size, size distribution, yield, EE, and LC) were investigated. The stability of the freeze‐dried phytosomes, a suitable state for long‐term storage of a food ingredient or nutraceutical, was assessed by their exposure to combinations of different relative humidities (RH; 7%, 53%, and 75%) and storage temperatures (25 and 40°C).

## 2 MATERIALS AND METHODS

### 2.1 Chemicals

High‐performance liquid chromatography (HPLC)‐grade solvents (acetonitrile (MeCN), acetic acid, and dimethyl sulfoxide (DMSO)) and chloroform (CHCl_3_, anhydrous, 99.9%) were obtained from Sigma‐Aldrich (St. Louis, MO, USA); ethanol (EtOH, 99.9%) from Kimix Chemical and Lab Supplies (Cape Town, South Africa); and Lipoid S 40 (PC, fat‐free soybean lecithin containing 40% phosphatidylcholine) from Brenntag (Johannesburg, South Africa). All other analytical‐grade chemicals used for physicochemical analyses and storage were obtained from Sigma‐Aldrich. Authentic reference standards for identification and quantification were obtained from Phytolab (Vestenbergsgreuth, Germany; mangiferin, vicenin‐2, luteolin, and 3‐β‐D‐glucopyranosyliriflophenone (IMG)), Extrasynthese (Genay, France; eriocitrin), and Sigma‐Aldrich (hesperidin). Deionized water was prepared using an Elix water purification system which was further purified to HPLC grade using a Milli‐Q academic water purification system from Merck (Darmstadt, Germany).

### 2.2 Preparation of CSE

The extract was prepared as previously described by Human et al. (2021). *Cyclopia subternata* plants were harvested from a trial plantation block, established by the Crop Development Division of ARC Infruitec‐Nietvoorbij on Nietvoorbij Research Farm from seeds obtained from the ARC honeybush gene bank. Briefly, freshly harvested *C. subternata* shoots (6.1 kg) were mechanically shredded (≤3 mm), dried at 40°C in a drying tunnel, and coarsely milled, using a Retsch rotary mill (Haan, Germany) equipped with a 1 mm sieve. The milled plant material (3 × 120 g) was extracted for 30 min, using aqueous EtOH (40%) in a 1:10 ratio (m/v). The process was repeated three times whereafter the extracts were pooled and filtered, followed by rotary evaporation of the EtOH under vacuum at 40°C (Rotavap, Büchi Labortechnik AG, Flawil, Switzerland). The residue was suspended in water, freeze‐dried (Virtis freeze‐drier, SP Scientific, Warminster, PA, USA), and stored in a sealed container until further use. The phenolic content of the extract was determined using HPLC coupled with diode array detection (DAD; refer to Section 2.4).

### 2.3 Preparation of CSE‐NVs

The nano‐phytosomes were prepared using the solvent evaporation method as previously described by Babazadeh et al. (2017) with adjustments. CSE‐NV production was initiated by dissolving CSE and PC in 90% aqueous EtOH (200 mL, 2.4 mg/mL solids) by sonication for 5 min (Bransonic, Danbury, CT, USA, 250 W, 44 kHz). The mixture was stirred for 2 h in a jacketed vessel heated using a circulating water bath (FMH Electronics, Cape Town, South Africa). After completion of the reaction, the solvent was evaporated under rotary vacuum evaporation to form a thin film. This film was suspended in deionized water (40 mL, 12 mg/mL solids), sonicated on ice using a Vibra‐cell probe sonicator (Sonics, Newtown, CT, USA) in pulse mode (1 s on/off cycle), and freeze‐dried.

Following preliminary testing, three process conditions were varied according to a central composite design (CCD): reaction and evaporation temperature (25–65°C, $X_{1}$), probe sonication time (0–60 min, $X_{2}$), and PC:CSE ratio (1.5–9:1 (%, m/m), $X_{3}$). The CCD consisted of 20 experimental runs performed in triplicate in a randomized order (Table 1). The effects of these variables were determined for the following responses: vesicle size (nm), polydispersity index (PDI), zeta potential (mV), reaction yield (%), EE (%), and LC (%). The results from the CCD runs were used to select a single set of suitable processing conditions for physicochemical analyses and stability testing.

**TABLE 1:** Process parameter levels (coded and de‐coded reaction and evaporation temperature, sonication time, and fat‐free soybean lecithin [PC]: green *Cyclopia subternata* extract [CSE] ratio) and responses (vesicle size, polydispersity index [PDI], zeta potential, yield, encapsulation efficiency [EE], and loading capacity [LC]) of a central composite design to produce green *Cyclopia subternata* extract nano‐phytosome vesicles (CSE‐NV).

Run number	Coded levels			De‐coded levels			Responses (average ± SD, n = 3)					
	X1	X2	X3	Temperature (°C)	Sonication time (min)	PC:CSE (%, m/m)	Size (nm)	PDI	Zeta potential (mV)	Yield	EE (%)	LC (%)
	Temperature (°C)		PC:CSE (%, m/m)									
1 (F)^a^	−1	−1	−1	33	12	5.2	114 ± 4	0.33 ± 0.04	−46.4 ± 2.2	95.2 ± 0.6	69.9 ± 0.6	11.0 ± 0.2
2 (F)	−1	−1	1	33	12	1.9	138 ± 4	0.32 ± 0.04	−42.3 ± 0.5	96.0 ± 2.4	12.5 ± 4.4	3.9 ± 1.5
3 (F)	−1	1	−1	33	48	5.2	151 ± 15	0.47 ± 0.06	−45.3 ± 4.6	95.3 ± 0.7	75.9 ± 2.2	11.9 ± 0.3
4 (F)	−1	1	1	33	48	1.9	177 ± 5	0.41 ± 0.03	−42.1 ± 2.6	94.9 ± 0.2	8.8 ± 0.2	2.8 ± 0.1
5 (F)	1	−1	−1	57	12	5.2	129 ± 4	0.35 ± 0.00	−40.9 ± 2.0	95.4 ± 0.0	72.7 ± 1.4	11.4 ± 0.3
6 (F)	1	−1	1	57	12	1.9	179 ± 7	0.35 ± 0.04	−41.3 ± 0.3	94.3 ± 0.3	11.5 ± 0.2	3.6 ± 0.1
7 (F)	1	1	−1	57	48	5.2	116 ± 30	0.47 ± 0.03	−40.8 ± 3.3	94.5 ± 1.0	73.1 ± 1.8	11.5 ± 0.3
8 (F)	1	1	1	57	48	1.9	158 ± 2	0.35 ± 0.01	−40.3 ± 2.3	94.2 ± 0.2	11.5 ± 0.4	3.6 ± 0.1
9 (S)^b^	−1.682	0	0	25	30	3.0	138 ± 3	0.42 ± 0.06	−44.3 ± 1.8	94.5 ± 0.9	56.1 ± 0.6	13.5 ± 0.1
10 (S)	1.682	0	0	65	30	3.0	135 ± 5	0.39 ± 0.01	−38.9 ± 2.4	93.5 ± 0.7	58.8 ± 8.8	14.1 ± 2.2
11 (S)	0	−1.682	0	45	0	3.0	313 ± 16	0.44 ± 0.02	−53.6 ± 1.6	94.2 ± 0.8	51.7 ± 1.1	12.4 ± 0.4
12 (S)	0	1.682	0	45	60	3.0	144 ± 3	0.48 ± 0.05	−42.3 ± 1.3	94.1 ± 0.5	61.2 ± 1.2	14.7 ± 0.1
13 (S)	0	0	−1.682	45	30	9.0	116 ± 2	0.45 ± 0.05	−45.8 ± 2.9	94.0 ± 0.8	79.0 ± 2.1	7.8 ± 0.2
14 (S)	0	0	1.682	45	30	1.5	206 ± 8	0.35 ± 0.01	−40.7 ± 0.8	93.8 ± 0.6	3.7 ± 0.1	1.3 ± 0.0
15 (C)^c^	0	0	0	45	30	3.0	145 ± 4	0.38 ± 0.03	−43.7 ± 2.9	94.0 ± 2.1	43.8 ± 0.4	10.5 ± 0.1
16 (C)	0	0	0	45	30	3.0	137 ± 0	0.31 ± 0.00	−41.0 ± 1.2	94.7 ± 1.7	47.2 ± 2.0	11.3 ± 0.3
17 (C)	0	0	0	45	30	3.0	137 ± 2	0.40 ± 0.07	−39.8 ± 1.0	95.1 ± 1.4	50.5 ± 1.0	12.1 ± 0.4
18 (C)	0	0	0	45	30	3.0	138 ± 2	0.33 ± 0.01	−40.9 ± 0.3	95.7 ± 1.2	43.6 ± 0.8	10.5 ± 0.3
19 (C)	0	0	0	45	30	3.0	131 ± 2	0.33 ± 0.02	−40.3 ± 2.4	94.4 ± 1.1	51.2 ± 1.1	12.4 ± 0.4
20 (C)	0	0	0	45	30	3.0	145 ± 1	0.31 ± 0.00	−41.3 ± 1.8	95.3 ± 0.6	51.2 ± 0.3	12.3 ± 0.2
CSE‐NV produced at selected conditions with favorable responses				40	32	4.9	127 ± 2	0.40 ± 0.02	−40. ± 1.6	96.2 ± 0.5	71.0 ± 5.5	11.9 ± 0.9

^a^ Factorial design point.

^b^ Star point.

^c^ Central point.

#### 2.3.1 Evaluation of CSE‐NV

##### Size, PDI, and zeta potential of CSE‐NV

The freeze‐dried phytosomes were suspended in distilled water (1 mg/mL) and photon correlation spectroscopy (Zetasizer Nanoseries 2590, Malvern Instruments, Worcestershire, England) was performed to determine the size of the phytosomes. The PDI was used to describe their size distribution. The zeta potential was calculated automatically using the electrophoretic mobility of the phytosomes in solution. Measurements were repeated three times with each measurement consisting of four scans at 25°C.

##### Yield, EE, and LC of CSE‐NV

Product yield was determined gravimetrically and expressed as a percentage of the sum of the mass of CSE and PC used for nano‐phytosome preparation (Equation 1).

(1) $$\frac{\text{mass of}\;\mathrm{CSE}\;\text{and}\;\mathrm{PC}\;\text{initially added}}{\text{mass of freeze}-\text{dried}\;\mathrm{CSE}-\mathrm{NV}\;\text{obtained}}\times 100$$

The phenolic content of CSE as determined by HPLC‐DAD (Section 2.4) was used to calculate the EE and LC. Given that only the CSE‐NV complex is soluble in CHCl_3_, the phenolic compounds bound to PC could be separated from those that were not bound. This entailed dissolving an aliquot of the phytosomes of each CCD run (*n* = 3 replicates) in CHCl_3_ (ca 30 mg in 15 mL, 2 mg/mL), vortexing and filtering through 0.45‐μm‐pore‐size Millex‐HV syringe filters (Millipore‐Sigma, Burlington, MA, USA). A set volume of the filtrate (4 mL) was dried using a SpeedVac concentrator (Savant SPD2010, Thermo Fisher Scientific, Waltham, MA, USA). The dried residue was dispersed in 2 mL 10% (v/v) aqueous DMSO by sonication (Bransonic, 250 W, 44 kHz; 15 min). Ascorbic acid (ca 9 mg/mL, final concentration) was added before sonication to prevent degradation of the phenolic compounds. Aliquots of the sonicated samples were frozen until HPLC analysis was performed (detailed in Section 2.4).

Only the major compounds that could be quantified in the extract were taken into account to calculate the sum of the individual phenolic content values ($\sum_{\mathrm{QP}}$) used to calculate the EE and LC. For calculation of $\sum_{\mathrm{QP}}$, the following phenolic compounds were quantified: 3‐β‐D‐glucopyranosyl‐4‐*O*‐β‐D‐glucopyranosyliriflophenone (IDG); IMG; 3′,5′‐di‐β‐D‐glucopyranosyl‐3‐hydroxyphloretin (HPDG); 3′,5′‐di‐β‐D‐glucopyranosylphloretin (PDG); and hesperidin, mangiferin, isomangiferin, vicenin‐2, and scolymoside. The EE was calculated by expressing the mass of the encapsulated phenolic compounds (g) as a percentage of the $\sum_{\mathrm{QP}}$ (g) initially added to the nano‐phytosome preparation as part of CSE (Equation 2). The EE for individual phenolic compounds was determined in the same manner (Equation 3). The LC was calculated by expressing the $\sum_{\mathrm{QP}}$ (g) as a percentage of the mass of CSE‐NV (g, Equation 4).

(2) $$\frac{\text{mass}\;\left(\sum_{\mathrm{QP}}\;\text{in}\;\mathrm{CSE}\;\text{initially added}\right)}{\text{mass}\;\left(\sum_{\mathrm{QP}}\;\text{in}\;\mathrm{CSE}-\mathrm{NV}\right)}\times 100$$

(3) $$\frac{\text{mass}\;\left(Phenolic\;compoud\;\text{in}\;\mathrm{CSE}\;\text{initially added}\right)}{\text{mass}\;\left(Phenolic\;compoud\;\text{in}\;\mathrm{CSE}-\mathrm{NV}\right)}\times 100$$

(4) $$\frac{\text{mass}\;\left(\sum_{\mathrm{QP}}\;\text{in}\;\mathrm{CSE}-\mathrm{NV}\right)}{\;\text{mass}\;\mathrm{CSE}-\mathrm{NV}}\times 100$$

##### Scanning electron microscopy (SEM) imaging of CSE‐NV

SEM images of the freeze‐dried phytosomes, prepared using the selected process conditions, were captured. SEM images of these phytosomes, re‐suspended in water, were also collected. Additionally, the phytosomes, produced using the CCD process conditions at three design points, were also resuspended in water and SEM images were captured.

The CSE‐NVs were imaged using a Zeiss MERLIN field‐emission SEM (Carl Zeiss Microscopy, Jena, Germany). Suspended phytosomes (1 mg/mL in deonized water) were imaged after being dripped onto aluminum SEM stubs and dried in a fume hood. The freeze‐dried phytosomes were imaged by placing the dry material directly on SEM stubs. All samples were coated with a 10 nm gold layer (Leica EM ACE200, Wetzlar, Germany). For image analysis, the beam conditions were set at 5 kV acceleration voltage, 250 pA beam current (I‐Probe), and 3–5.5 mm working distance (WD). Images were captured using a Zeiss inlens (secondary electron) detector.

### 2.4 HPLC quantification of phenolic compounds in CSE and CSE‐NV

The phenolic content of CSE and CSE‐NV was determined using a previously validated method for *C. subternata* (De Beer et al., 2012). In brief, the equipment consisted of an Agilent 1200 HPLC System (Agilent Technologies, Santa Clara, CA, USA) with a DAD. Gradient separation was achieved on a Gemini‐NX C_18_ (150 × 4.6 mm; 3 μm; 110 Å) column (Phenomenex, Torrance, CA, USA) at a flow rate of 1 mL/min at 30°C with aqueous acetic acid (2%) and MeCN as mobile phases. The phenolic compounds were quantified (expressed as g/100 g extract) either at 288 nm or 320 nm using a 7‐point calibration curve prepared with authentic reference standards (mangiferin, vicenin‐2, luteolin, IMG, eriocitrin, and hesperidin). When reference standards were not available, response factors were used as described by Human et al. (2021). Briefly, isomangiferin and scolymoside were quantified based on response factors relative to mangiferin and luteolin and IDG, PDG, and HPDG from response factors relative to hesperidin. Before injection, standards and samples were filtered using 0.22‐μm‐pore‐size Millex‐HV syringe filters (Merck).

### 2.5 Physicochemical analysis of CSE‐NV, CSE, and PC

#### 2.5.1 Differential scanning calorimetry (DSC)

DSC analysis of the samples was performed using a Perkin Elmer DSC 8000 calorimeter (Waltham, MA, USA) equipped with Pyris Software (version 13.3.1.0014). The samples (ca 2 mg) were directly weighed into an aluminum pan and sealed. All analyses were conducted at a heating rate of 10°C/min and a dry nitrogen gas purge at a flow rate of 20 mL/min was applied.

#### 2.5.2 Fourier transform infrared (FTIR)

FTIR analysis was performed using a Perkin Elmer Spectrum 400 FTIR spectrometer equipped with a diamond attenuated total reflectance (ATR) crystal and spectrum software (version 6.3.5.017). The sample, placed on the sample stage, was scanned over 650–4000 cm^−1^ at 2 cm^−1^ resolution.

#### 2.5.3 X‐ray powder diffraction (XRPD)

XRPD data were collected using a Bruker D8 Advance powder X‐ray diffractometer (Karlsruhe, Germany). High voltage and current were set to 40 kV and 40 mA, respectively. All diffraction runs were performed at ambient temperature using a diffraction range of 4–40° 2θ and a scan rate of 0.1°/min.

#### 2.5.4 Nuclear magnetic resonance (NMR)

Solutions of CSE and PC (0.07 mg/μL) and CSE‐NV (0.03 mg/μL) were prepared in deuterated chloroform (CDCl_3_). Each solution was thoroughly mixed followed by filtering through a 0.2 μm PVDF filter directly into an NMR vial for subsequent analysis.

NMR measurements were performed on a Bruker 400 MHz AVANCE III HD Nanobay NMR spectrometer (Billerica, MA, USA) equipped with a 5 mm BBI probe at 298 K. ^1^H NMR spectra were recorded using a standard one‐pulse Bruker sequence with a spectral width of 16 ppm, centered at 4.7 ppm, 32 scans, 32 K data points, and a relaxation time fixed to 1 s. The residual CHCl_3_ signal was calibrated at 7.25 ppm.

NMR spectra of CSE and PC subjected to the selected process conditions for CSE‐NV preparation were recorded with a Bruker 600 MHz AVANCE NMR using the same conditions (different equipment was used due to equipment availability). This was done to confirm that the components are not chemically altered by the process and that the changes observed in the NMR spectra of CSE‐NV were indeed due to chemical interaction (Figure S1).

#### 2.5.5 Moisture content (MC), water activity (a_w_), and moisture sorption isotherm (MSI)

The MC (ca 3 g) was determined gravimetrically, using an HR73 Halogen Moisture Analyzer (Mettler Toledo, Greifensee, Switzerland). The samples were dried at 100°C for 60 min and the MC (wet basis) was converted to MC (dry basis). The a_w_ was measured at 25°C, using a Novasina LabMaster‐a_w_ electric hydrometer (Lachen, Switzerland).

Moisture sorption analyses were performed using a Q5000 Vapor Sorption Analyzer (TA Instruments, New Castle, DE, USA). The temperature was controlled at 25°C with the first humidity ramp set from 0% to 90% relative humidity (RH). Subsequent desorption was set to 90–10% RH. Each run started with a drying phase at 60°C for 60 min or until the weight fluctuation was less than 0.0001%. The MSI data were fitted to Brunauer–Emmet–Teller (BET) and Guggenheim–Anderson–de Boer (GAB) models. Based on the coefficient of determination (R_2_) values and visual observation (Table S1 and Figure S2), the GAB model provided the best fit and was used to calculate the monolayer moisture content (M_0_) (Labuza & Altunakar, 2007).

### 2.6 Powder stability testing

Stability testing of CSE and CSE‐NV, prepared according to the selected process conditions, was performed by storing the samples at 25°C and 40°C, exposed to ca 7%, 53%, and 75% RH using mini‐hygrostats. These consisted of 24 mL screw‐cap glass vials, each with a conical glass insert containing 250 μL of a saturated salt solution (Greenspan, 1977; Table S2), mounted in a stainless‐steel compression spring. Each vial was prepared by accurately weighing 80 mg sample directly into the vial, whereafter the spring was added and the total mass recorded. The glass inserts containing the salt solution were then placed in the spring. This suspended the insert above the powder.

The mini‐hygrostats were placed inside stability cabinets (SMC Scientific Manufacturing cc., Table View, South Africa) controlled to the required temperatures. At each time point, the sealed vials were removed from the stability cabinets, the glass inserts were removed, and the vials containing the powder and spring were weighed to determine the increase/decrease in powder mass due to moisture uptake/loss. The samples were prepared for HPLC analysis (Section 2.4) by dissolving the powder directly in the glass vial with 10 mL 10% (v/v) aqueous DMSO, followed by sonication for ca 15 min or until completely dispersed.

### 2.7 Statistical analysis

#### 2.7.1 CCD

The CCD data were analyzed using Statistica 13.0 (Statsoft Southern Africa, Sandton, South Africa). The statistical significance and suitability of the regression model, its factors, and their interactions were determined at the 5% probability level (*p* < 0.05) using univariate analysis of variance (ANOVA). The fitting efficiency of the data to the model was evaluated by calculating the *R* ^2^, adjusted *R* ^2^ (*R* ^2^_adj_), and the significance of lack‐of‐fit (LOF) values (Table S3). The normality of the data was visually evaluated by plotting the normal probability plots of the residuals (Figure S3).

**FIGURE 1:**
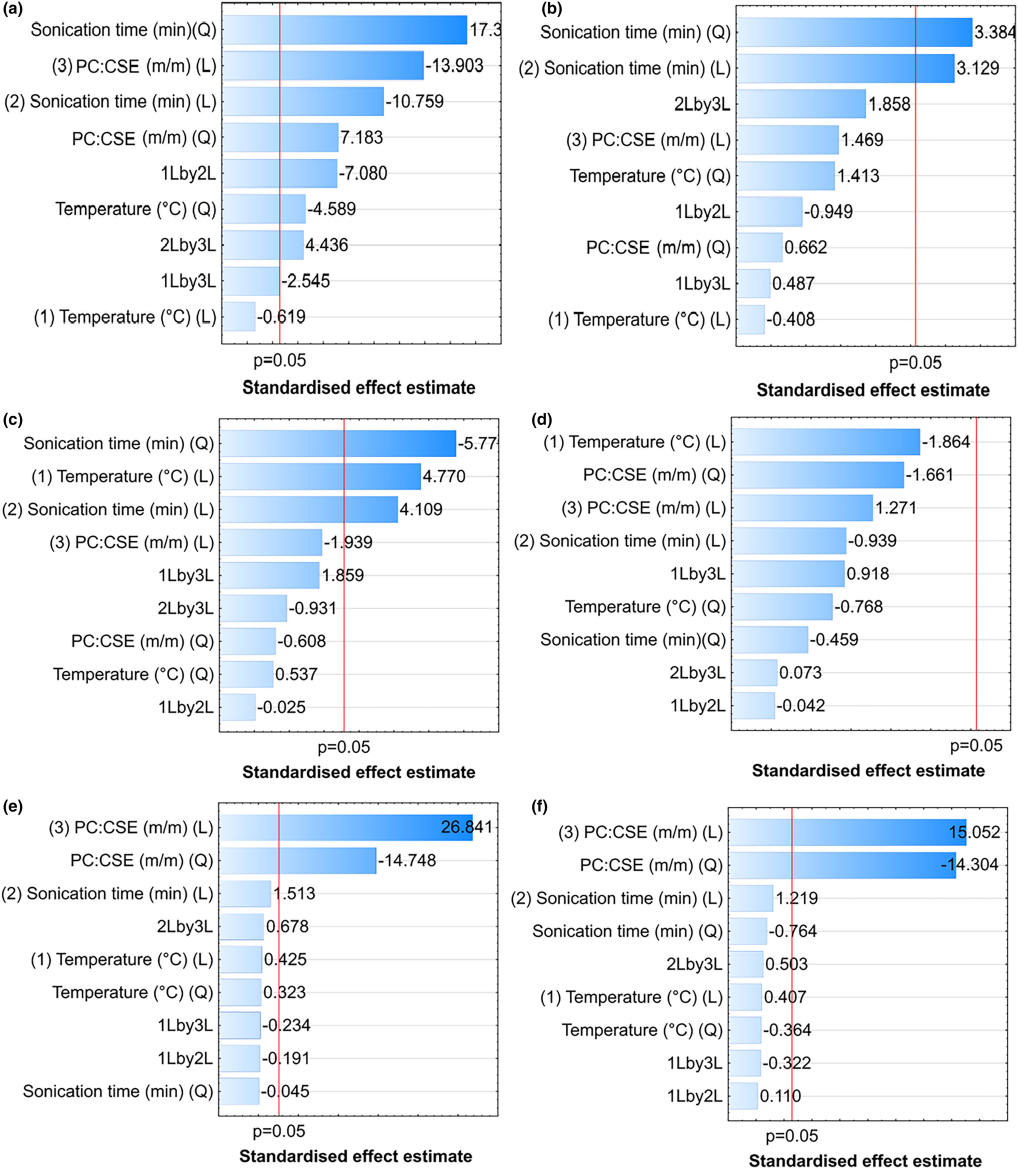
Standardized Pareto charts showing linear (L), quadratic (Q), and interaction effects of the temperature (°C), sonication time (min), and PC:CSE (%, m/m) ratio for (a) vesicle size, (b) polydispersity index, (c) zeta potential, (d) yield, (e) encapsulation efficiency, and (f) loading capacity. Process parameters and interactions crossing the red line were considered significant (*p* < 0.05). PC is fat‐free soybean lecithin containing 40% phosphatidylcholine and CSE is a *Cyclopia subternata* extract.

**FIGURE 2:**
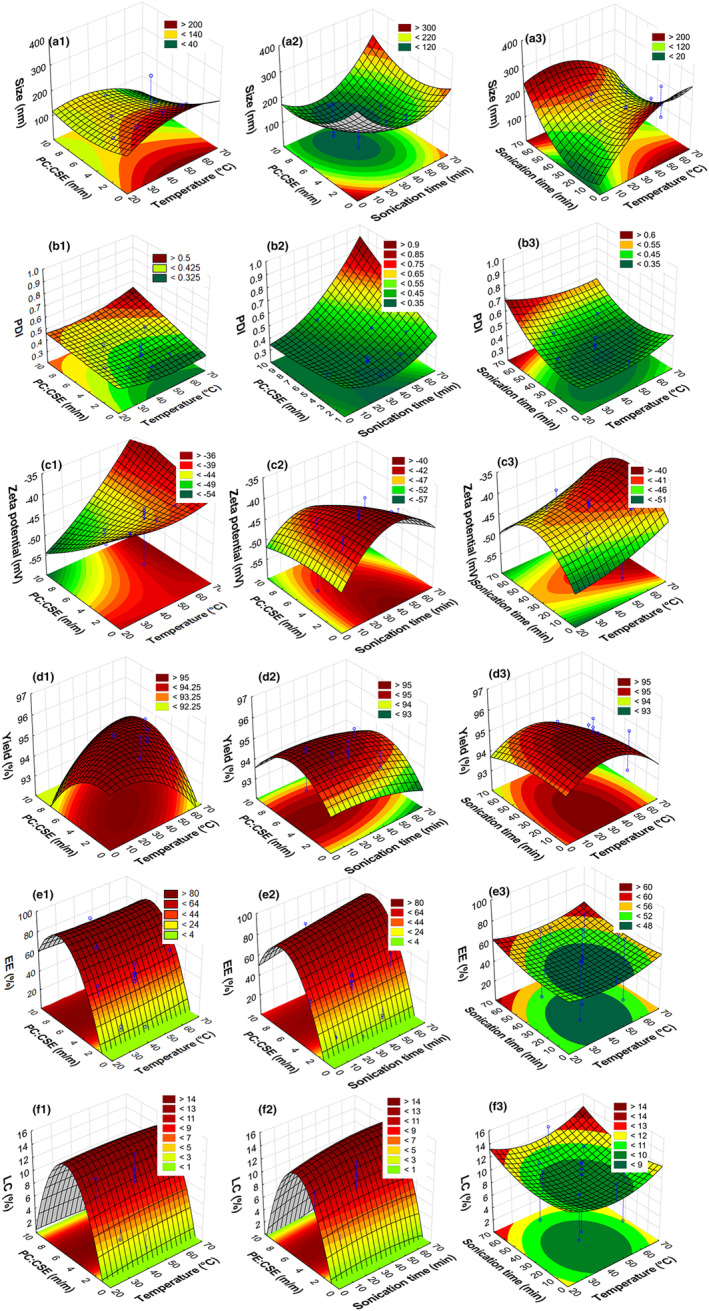
Combined response surface and contour plots showing effects of (1) PC:CSE ratio and temperature; (2) PC:CSE ratio and sonication time, and (3) sonication time and temperature on the (a) vesicle size, (b) polydispersity index (PDI), (c) zeta potential, (d) yield, (e) encapsulation efficiency (EE), and (f) loading capacity (LC). PC is fat‐free soybean lecithin containing 40% phosphatidylcholine and CSE is green *Cyclopia subternata* extract.

Standardized Pareto charts (Figure 1), two‐dimensional contour, and three‐dimensional response surface plots (Figure 2) were constructed.

#### 2.7.2 Powder stability testing

For each of the random replicates (*n* = 3) of the formulations (CSE and CSE‐NV), samples were prepared for each sampling time point (*n* = 5, plus an initial time point) and condition (*n* = 6), amounting to 180 mini‐hygrostats in total. Aliquots (*n* = 60) of experimental replicates of CSE and CSE‐NV were randomly assigned to the different temperature–RH combinations.

Zero‐order, first‐order, second‐order, and fractional conversion reaction kinetics models were fitted over the time points of each replicate formulation under the different storage conditions, using Proc NLIN of SAS software (Version 9.4; SAS Institute Inc, Cary, CA, USA). The second‐order model (Van Boekel, 2008) (Equation 5) gave the best fit based on *R* ^2^_adj_ values and was thus selected to predict compound degradation (Table S4 and Figures S4–S6).

(5) $$C=C_{0}/\left(1+C_{0}\mathrm{Kt}\right)$$

where *C* is the content of the phenolic compound (g/100 g extract) after time *t*, *C*_0_ is the initial content of the phenolic compound (g/100 g), *t* is the heating time in days, and *K* is the reaction rate constant.

ANOVA was performed on the regression parameters to determine significant effects, using the GLM (general linear models) procedure of SAS software. The Shapiro–Wilk test was performed on the residuals from the models to test for normality. Fisher's least significant difference was calculated at the 5% level to compare means where *p* < 0.05 was considered significant.

## 3 RESULTS

### 3.1 Modeling of CCD data

**FIGURE 3:**
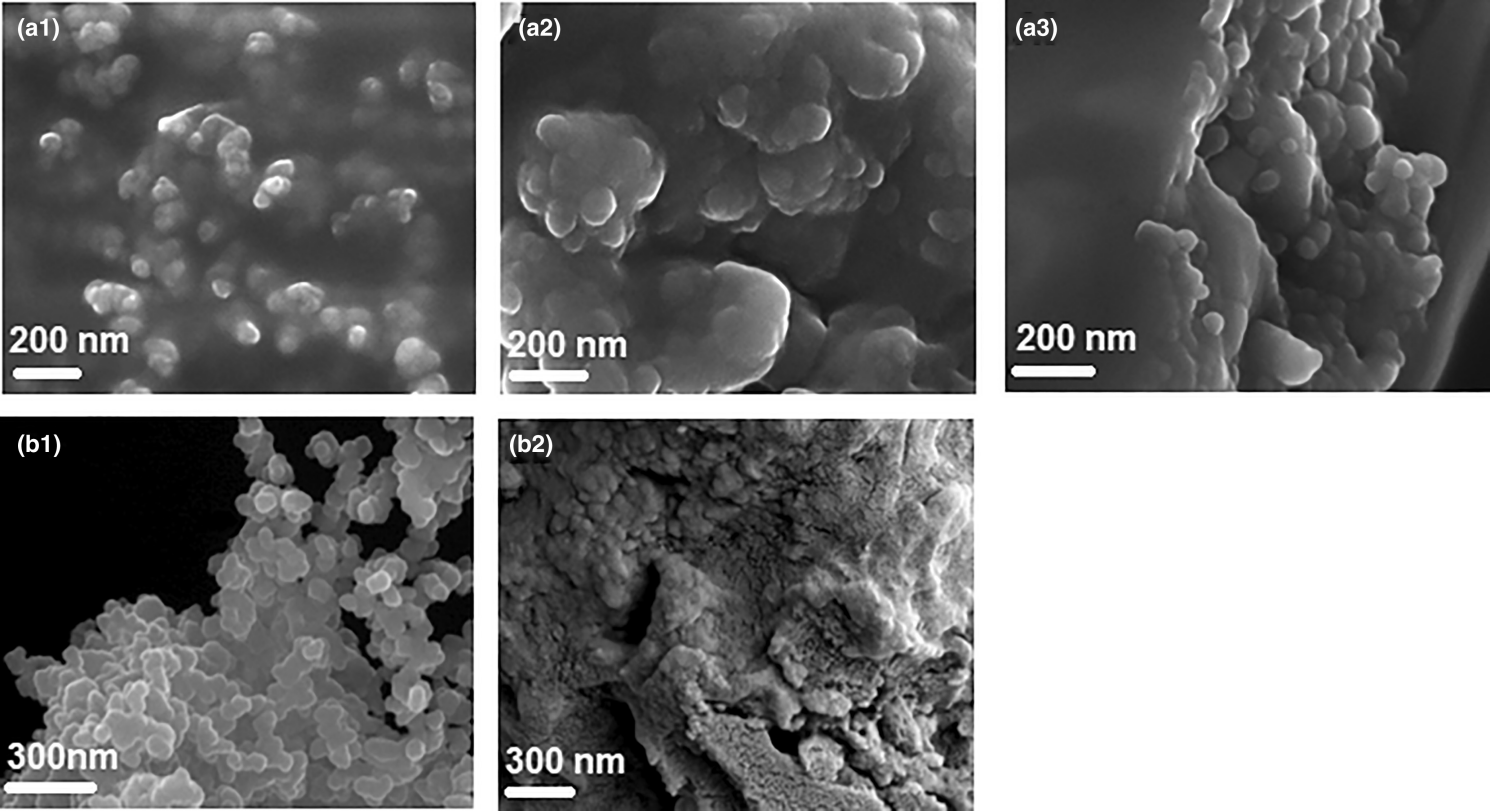
Scanning electron microscopy images of *Cyclopia subternata* extract nano‐phytosome vesicles (CSE‐NV) of a central composite design after freeze‐drying and re‐suspension in deionized water: (a1) Run 2 (factorial point), (a2) Run 4 (factorial point), (a3) Run 12 (star point), (b1) selected CSE‐NV formulation with favorable properties, and (b2) freeze‐dried powder of selected CSE‐NV formulation with favorable properties.

The results for yield, phytosome size, PDI, EE, and LC, as a function of variation in the formulation and process parameters, that is, the reaction temperature, sonication time, and ratio of PC to CSE, are summarized in Table 1. The formation of semi‐spherical nano‐sized phytosomes for selected runs (run 2, 4 (factorial), and 12 (star)) was confirmed by SEM analysis (Figure 3a1–a3). The re‐formation of nano‐sized semi‐spherical vesicles when the freeze‐dried powders, which showed a sheet‐like morphology, were re‐suspended in water was also confirmed (Figure 3b1,b2).

A polynomial regression model was fitted to the CCD response data. The fit of the models, evaluated in terms of R^2^, R^2^_adj_, and LOF (Table S3) (Bezerra et al., 2008), ranged between 0.4108–0.8964, 0.2554–0.8033, and 0.000079–0.3091, respectively, indicating reasonable‐to‐poor model fittings and non‐significant and significant LOF values. The poor fit of some of the models indicates that the measured properties will naturally vary to a small extent, independent of the selected process parameters, and can only be marginally controlled and predicted by adjusting the selected process parameters. These vesicle properties could also be dependent on other unknown process parameters not investigated in the current study. However, residuals for these properties appeared to be distributed with positive and negative deviations in the same proportion (Figure S3), indicating independent and negligible errors (Lee et al., 2011). This suggests the reliability of the data and their usefulness for further investigation to establish broader trends.

### 3.2 Effect of formulation and process parameters on physical properties of CSE‐NV

The significant effects of the process parameters on the different responses were illustrated by standardized Pareto charts (Figure 1), two‐dimensional contour, and three‐dimensional response surface plots (Figure 2).

The vesicle size varied between 113.7 and 312.7 nm and was significantly (*p* < 0.05) affected by almost all the linear, quadratic, and interaction effects of the process parameters with sonication time having the largest impact (Figure 1a). Figures 2a2 and 2a3 showed a decrease in vesicle size with sonication times between 0 and 35 min, followed by an increase between 35 and 70 min. The PDI varied between 0.31 and 0.48 and was only significantly (*p* < 0.05) affected by the sonication time (Figure 1b), since it increased with increasing sonication time (Figure 2b2, b3). The temperature and PC:CSE ratio had minimal effect on the vesicle size and PDI (Figure 2a1, b1). The zeta potential varied between −55.0 and −38.9 mV, where the quadratic and linear effects of the sonication time and the linear effect of temperature significantly (P < 0.05) affected this property (Figure 1c). Figure 2c1–c3 indicated that the highest absolute zeta potential can be obtained at lower temperatures (ca <40–50°C) and either shorter (ca < 30 min) or very long (ca > 60 min) sonication times.

Not only were high process yields (>93.5%) obtained but the yield was not significantly (*p* < 0.05) affected by the investigated process parameters (Figures 1d and 2d1‐d3).

The average EE and LC (based on the $\sum_{\mathrm{QP}}$) varied between 3.7% and 79.0% and 1.3% and 14.7%, respectively. The variation in the PC:CSE ratio was the sole cause of this significant variation in EE and LC (Figure 1e,f). The domed RSM plots (Figure 2e1,e2,f1,f2) clearly showed the quadratic effect of the PC:CSE ratio, where an increase in EE and LC is observed between PC:CSE ratios of ca 0–5:1, with a maximum at ca 4.9:1 and a decrease at higher ratios. The temperature and sonication time had no significant effect on the EE or LC (Figure 2e3, f3). The EE of the individual phenolic compounds varied (Table 2) and differed from the EE, based on $\sum_{\mathrm{QP}}$. For all CCD runs, IMG and IDG showed the highest and lowest EE (%) for phosphatidylcholine, respectively (Table 2).

**TABLE 2:** Process parameter levels (reaction and evaporation temperature, sonication time, and fat‐free soybean lecithin (PC):green *Cyclopia subternata* extract (CSE) ratio) and encapsulation efficiency (EE, *n* = 3, average ± SD) of individual phenolic compounds of a central composite design to produce *Cyclopia subternata* extract nano‐phytosome vesicles (CSE‐NV).

Run number	Process parameters			Phenolic compounds EE (%)									
	Temperature (°C)	Sonication time (min)	PC:CSE (%, m/m)	IDG^a^	IMG^b^	HPDG^c^	Eriocitrin	PDG^d^	Hesperidin	Mangiferin	Isomangiferin	Vicenin‐2	Scolymoside
1 (F)^e^	33	12	5.2	61.3 ± 1.5	72.2 ± 1.2	74.5 ± 1.2	71.2 ± 0.1	76.6 ± 1.3	74.3 ± 1.1	69.4 ± 0.8	67.7 ± 0.7	69.3 ± 0.8	62.1 ± 1.4
2 (F)	33	12	1.9	7.4 ± 3.3	23.8 ± 4.2	15.9 ± 5.5	15.8 ± 4.9	17.6 ± 5.1	21.3 ± 3.9	12.1 ± 4.5	14.5 ± 4.3	11.0 ± 4.2	10.7 ± 4
3 (F)	33	48	5.2	63.0 ± 2.7	78.9 ± 1.6	78.3 ± 1.4	81.6 ± 2.9	82.5 ± 1.7	81.5 ± 1.5	76.5 ± 3.1	72.3 ± 3.1	73.8 ± 2.8	70.3 ± 2.3
4 (F)	33	48	1.9	3.7 ± 0.3	15.2 ± 0.4	8.6 ± 0.3	8.7 ± 0.2	9.9 ± 0.2	13.7 ± 0.3	7.1 ± 0.2	8.7 ± 0.2	6.4 ± 0.2	5.8 ± 0.1
5 (F)	57	12	5.2	61.0 ± 0.7	75.7 ± 1.2	75.6 ± 2.5	76.3 ± 1.5	78.3 ± 1.2	76.7 ± 1.2	72.5 ± 2.1	71.7 ± 1.8	73.9 ± 1.3	65.2 ± 2.8
6 (F)	57	12	1.9	5.0 ± 0.3	19.0 ± 0.3	11.7 ± 0.4	11.9 ± 0.5	13.3 ± 0.5	17.4 ± 0.4	9.2 ± 0.3	11.4 ± 0.2	8.2 ± 0.4	7.9 ± 0.2
7 (F)	57	48	5.2	63.9 ± 5.7	76.9 ± 0.8	75.9 ± 1.3	76.5 ± 1.6	80.1 ± 0.9	78.1 ± 1.5	71.4 ± 0.8	68.8 ± 1.7	74.0 ± 3	65.1 ± 1.4
8 (F)	57	48	1.9	5.1 ± 0.3	19.9 ± 0.3	11.4 ± 0.3	11.9 ± 0.4	13.2 ± 0.4	17.9 ± 0.4	8.8 ± 0.3	11.2 ± 0.4	8.3 ± 0.7	7.7 ± 0.3
9 (S)^f^	25	30	3.0	40.0 ± 1.1	63.9 ± 2.2	62.7 ± 1	61.0 ± 1.1	64.9 ± 1.4	61.1 ± 1.5	54.1 ± 0.3	54.8 ± 0.9	51.5 ± 0.8	47.3 ± 0.3
10 (S)	65	30	3.0	35.7 ± 7.5	58.9 ± 9.9	59.9 ± 10	58.6 ± 9.2	61.8 ± 9.7	58.2 ± 9.2	53.0 ± 8.4	54.1 ± 8.5	51.0 ± 8.8	46.4 ± 7.1
11 (S)	45	0	3.0	33.7 ± 2.2	57.2 ± 0.6	58.8 ± 0.9	56.0 ± 0.6	60.1 ± 0.5	56.1 ± 0.7	51.0 ± 2	53.4 ± 1.1	46.0 ± 2.1	44.7 ± 1.1
12 (S)	45	60	3.0	39.9 ± 1.2	66.1 ± 1.8	69.8 ± 1.5	67.3 ± 2.1	71.4 ± 1.9	66.4 ± 1	60.7 ± 1	60.9 ± 1.1	55.5 ± 0.8	54.1 ± 1.1
13 (S)	45	30	9.0	74.9 ± 0.9	75.2 ± 2.3	81.4 ± 2.6	80.5 ± 2.8	84.1 ± 2.4	83.2 ± 1.5	76.9 ± 2	75.4 ± 2.7	86.1 ± 3	72.9 ± 2.4
14 (S)	45	30	1.5	0.8 ± 0.0	7.9 ± 0.5	2.5 ± 0.1	3.1 ± 0.1	3.7 ± 0.2	7.2 ± 0.6	2.3 ± 0.1	3.6 ± 0.1	3.7 ± 0.1	1.9 ± 0.0
15 (C)^g^	45	30	3.0	30.7 ± 2.1	51.3 ± 1.2	48.2 ± 0.3	47.0 ± 0.6	51.0 ± 0.2	50.7 ± 0.6	40.0 ± 1	42.6 ± 0.7	40.4 ± 0.5	35.8 ± 2.1
16 (C)	45	30	3.0	30.4 ± 2.3	53.4 ± 2.1	53.4 ± 2.6	50.8 ± 2.5	54.8 ± 2.5	53.4 ± 1.1	45.5 ± 3.9	47.7 ± 3.4	42.5 ± 1.5	40.2 ± 2.2
17 (C)	45	30	3.0	30.6 ± 1.4	57.1 ± 2.8	53.3 ± 7.5	55.8 ± 1.4	57.6 ± 2.2	55.4 ± 0.9	51.2 ± 1.9	53.1 ± 1.6	46.6 ± 0.8	44.1 ± 2.7
18 (C)	45	30	3.0	26.5 ± 0.1	54.0 ± 0.6	36.4 ± 1.3	49.1 ± 1.3	48.9 ± 0.7	50.1 ± 0.2	45.0 ± 2.3	48.0 ± 1.5	42.6 ± 0.3	35.9 ± 1.0
19 (C)	45	30	3.0	30.0 ± 1.3	60.2 ± 1.3	42.8 ± 2.1	58.4 ± 1.3	57.7 ± 1.8	56.7 ± 1.8	54.8 ± 1.4	57.4 ± 1.7	49.3 ± 1.3	45.1 ± 1.0
20 (C)	45	30	3.0	30.9 ± 0.7	61.2 ± 0.4	43.0 ± 0.4	58.5 ± 0.5	57.6 ± 0.4	57.6 ± 0.7	53.2 ± 1.0	56.2 ± 0.9	49.7 ± 2.5	44.4 ± 0.6
Minimum				0.8	7.9	2.5	3.1	3.7	7.2	2.3	3.6	3.7	1.9
Maximum				74.9	78.9	81.4	81.6	84.1	83.2	76.9	75.4	86.1	72.9
Average				33.7	52.4	48.2	50	52.3	51.8	45.7	46.7	44.5	40.4

^a^ 3‐β‐D‐Glucopyranosyl‐4‐*O*‐β‐D–glucopyranosyliriflophenone.

^b^ 3‐β‐D‐Glucopyranosyliriflophenone.

^c^ 3′,5′‐di‐β‐D‐Glucopyranosyl‐3‐hydroxyphloretin.

^d^ 3′,5′‐di‐β‐D‐Glucopyranosylphloretin.

^e^ Factorial design point.

^f^ Star point.

^g^ Central point.

For confirmation of the hydrogen bonding between phosphatidylcholine and the CSE phenolic compounds and their stability in powder form (Sections 3.3, 3.4, 3.5), CSE‐NV was produced using a fixed set of process conditions (Table 1: “CSE‐NV produced at set selected conditions with favourable responses”). Process conditions that resulted in higher yield and EE, as well as phytosomes with higher LC and zeta potential, smaller vesicle size, and PDI, were selected. At these set conditions, CSE‐NVs with LC, vesicle size, PDI, and zeta potential of 11.9 ± 0.9%, 126.6 ± 1.9 nm, 0.40 ± 0.02, and −40.9 ± 1.6 mV, respectively, were obtained. The yield and EE were 96.2 ± 0.5% and 71.0 ± 5.5%, respectively.

### 3.3 DSC, FTIR XRPD, and NMR of selected CSE‐NV formulation

**FIGURE 4:**
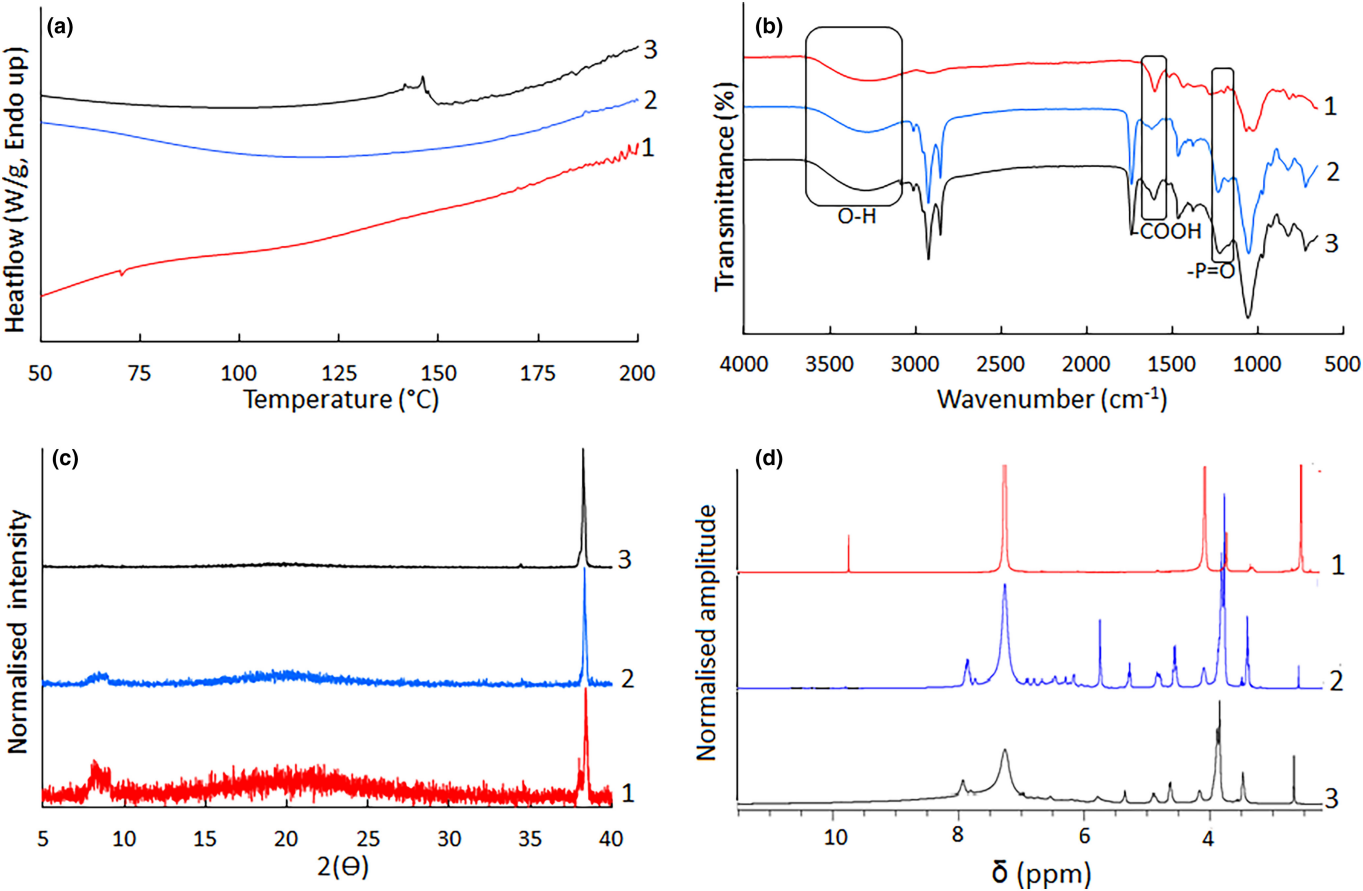
(a) Differential scanning calorimetry thermograms, (b) Fourier transform infrared spectra, (c) X‐ray powder diffractograms, and (d) ^1^H nuclear magnetic resonance for (1) green *Cyclopia subternata* extract (CSE), (2) fat‐free soybean lecithin containing 40% phosphatidylcholine (PC), and (3) green *C. subternata* extract nano‐phytosome vesicles (CSE‐NV).

FIGURE 4a depicts the DSC thermograms of PC, CSE, and CSE‐NV. The PC and CSE thermograms show no distinctive crystalline melting peaks and the CSE‐NV thermogram shows a small group of endothermic peaks between 140 and 150°C not present in the PC and CSE thermograms.

The FTIR spectra (Figure 4b) of PC and CSE‐NV depict characteristic C–H stretching signals at 2884 and 2850 cm^−1^ for the long fatty acid chain, a C=O stretching band at 1738 cm^−1^ for the fatty acid ester, and P=O stretching band at 1217 cm^−1^ for phosphatidylcholine (Saoji et al., 2016). The spectra of PC and CSE‐NV (Figure 4b) are very similar, with only minor differences that include a slight enlargement of the O–H band and deformation of the P=O stretching band at 3600–3100 cm^−1^ and 1217 cm^−1^, respectively.

The XRPD diffractograms show CSE, PC, and CSE‐NV to be largely amorphous. The distinct diffraction peak observed at 2θ = 38.4° for all the samples is attributed to diffraction from the sample holder used during the collection of the XRPD diffraction patterns (Figure 4c). The XRPD diffractograms of CSE and PC both exhibited a broad halo peak between 2θ = 8–9°, while that of CSE also showed a small peak at 2θ = 38.2°.

The NMR spectrum (Figure 4d) of PC showed distinct signals at ca 3.2–3.4 ppm and between 4–6 ppm. Compared to PC, these signals are broadened and suppressed in the CSE‐NV spectrum. The CSE spectrum showed distinct signals between 4–5 ppm and 9–10 ppm which are severely suppressed or not observed for CSE‐NV.

### 3.4 MSIs

**FIGURE 5:**
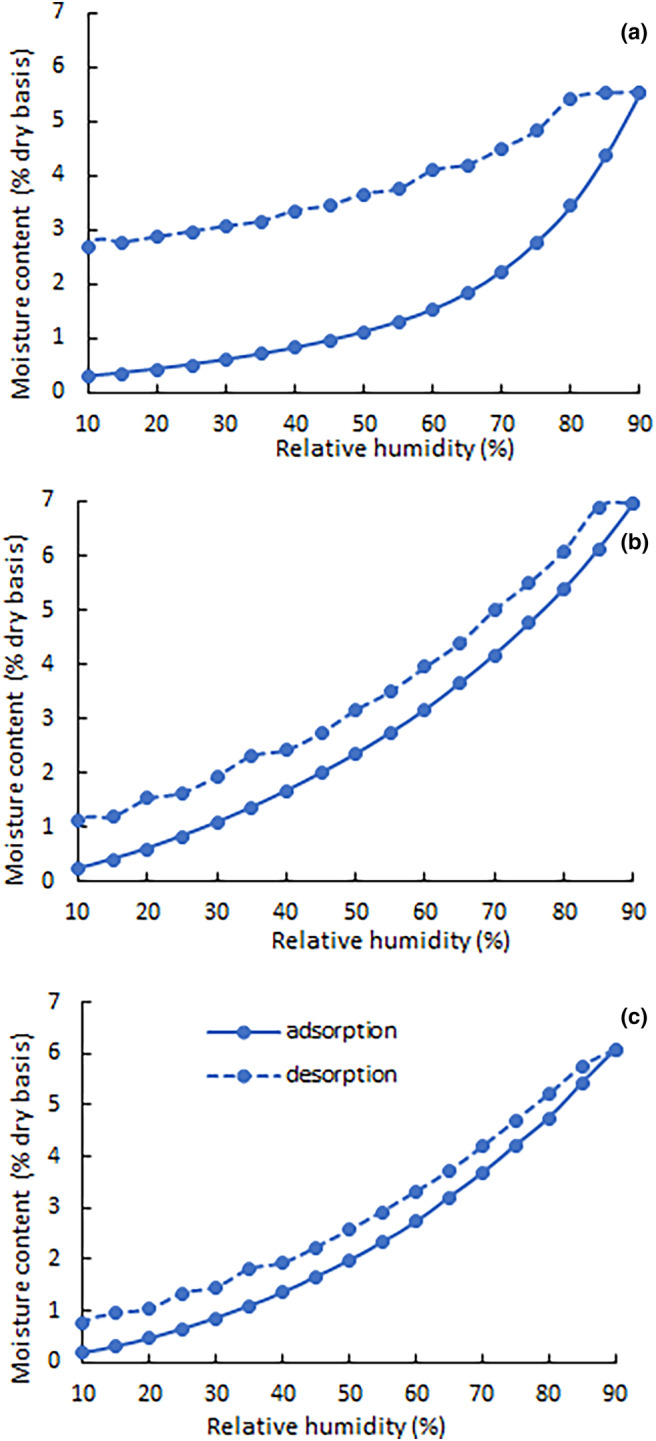
Moisture adsorption and desorption isotherms at 25°C for (a) green *Cyclopia subternata* extract (CSE), (b) fat‐free soybean lecithin containing 40% phosphatidylcholine (PC), and (c) green *C. subternata* nano‐phytosome vesicles (CSE‐NV).

The MSIs of CSE, PC, and CSE‐NV showed that the moisture uptake of their powders increased as the humidity increased (Figure 5). The MSI of CSE‐NV was more similar to that of PC than CSE. At the high RH levels, CSE‐NV may absorb more water than CSE (e.g., 2% vs. 1% at 50% RH for CSE‐NV and CSE, respectively). CSE‐NV, on the other hand, had a significantly smaller hysteresis loop than CSE.

The M_0_ values (Table 3), calculated from the GAB model for CSE, PC, and CSE‐NV, were 0.59%, 4.19%, and 3.94%, respectively.

**TABLE 3:** Moisture content (MC), water activity (*a*_w_), and monolayer moisture content (M_0_) for Guggenheim–Anderson–de Boer (GAB) model fitted to moisture adsorption data obtained at 25°C for a green *Cyclopia subternata* extract (CSE), fat‐free soybean lecithin (PC) containing 40% phosphatidylcholine, and green *Cyclopia subternata* extract nano‐phytosome vesicles (CSE‐NV).

Sample	MC (% dry basis)	*a*_w_	M_0_ (% dry basis)
CSE	6.65 ± 0.14	0.259 ± 0.002	0.59
PC	0.99 ± 0.03	0.062 ± 0.003	4.19
CSE‐NV	3.85 ± 0.05	0.338 ± 0.004	3.94

### 3.5 Stability trial

The effects of temperature and RH on the stability of the individual compounds were assessed using the second‐order reaction rate constants (K) (Table 4). IMG, included in the calculation of the EE, was not quantified for the stability study due to the suspected formation of compounds overlapping with its peak, which would result in an underestimation of its degradation.

**TABLE 4:** Reaction rate constants (*K*, *n* = 3, average ± SD) of the second‐order model^†^ fitted to the experimental data representing the degradation of the phenolic compounds and moisture uptake of a green *Cyclopia subternata* extract (CSE) and green *Cyclopia subternata* nano‐phytosome vesicles (CSE‐NV) during storage at different temperature and relative humidity (RH) combinations (ca 7, 53, and 75% RH at 25 and 40°C) for 180 days.

Compound	RH (%)	K ((g/100 g)^−1^ days^−1^) × 10^3^					
		7% RH		53% RH		75% RH	
	Sample	CSE	CSE‐NV	CSE	CSE‐NV	CSE	CSE‐NV
	Temperature (°C)						
IDG^‡^	25	0.333 ± 0.020c*	0.294 ± 0.097d	0.322 ± 0.039c	0.364 ± 0.054bc	0.382 ± 0.015bc	0.385 ± 0.077bc
	40	0.332 ± 0.017c	0.353 ± 0.094c	0.408 ± 0.020bc	0.570 ± 0.075a	0.460 ± 0.016b	0.659 ± 0.167a
HPDG^§^	25	1.490 ± 0.226f	0.811 ± 0.180g	1.731 ± 0.157ef	2.047 ± 0.219e	2.810 ± 0.106d	2.725 ± 0.393d
	40	1.865 ± 0.061ef	1.758 ± 0.424ef	3.483 ± 0.207c	5.416 ± 0.297b	8.878 ± 0.341a	8.598 ± 0.470a
PDG^¶^	25	0.487 ± 0.042f	0.322 ± 0.053g	0.550 ± 0.083ef	0.495 ± 0.068f	0.737 ± 0.020d	0.603 ± 0.106e
	40	0.536 ± 0.021ef	0.574 ± 0.057ef	0.884 ± 0.023c	1.378 ± 0.023b	2.071 ± 0.034a	1.391 ± 0.104b
Mangiferin	25	0.321 ± 0.018h*	0.200 ± 0.027i	0.347 ± 0.056h	0.464 ± 0.002g	0.974 ± 0.014e	1.427 ± 0.062d
	40	0.320 ± 0.009h	0.228 ± 0.085i	0.548 ± 0.021f	1.858 ± 0.056c	5.219 ± 0.020b	7.690 ± 0.125a
Isomangiferin	25	1.264 ± 0.067f	0.243 ± 0.126h	1.458 ± 0.255ef	0.787 ± 0.106g	1.780 ± 0.048cd	1.907 ± 0.274c
	40	1.225 ± 0.072f	0.611 ± 0.112g	1.590 ± 0.079de	2.403 ± 0.149b	4.962 ± 0.043a	5.163 ± 0.284a
Scolymoside	25	0.825 ± 0.041bc	0.528 ± 0.146f	0.761 ± 0.135cde	0.660 ± 0.028acde	0.885 ± 0.027b	0.609 ± 0.191ef
	40	0.769 ± 0.019cde	0.639 ± 0.046def	0.798 ± 0.033cd	0.595 ± 0.144ef	1.706 ± 0.077a	0.633 ± 0.253def
Moisture uptake (%, wet basis)	25	−1.05 ± 0.04f	−0.49 ± 0.1ef	4.90 ± 0.08d	4.51 ± 0.14d	12.43 ± 0.52a	10.28 ± 0.48b
	40	−1.79 ± 0.51g	0.10 ± 0.67e	5.81 ± 0.21c	4.78 ± 0.08d	13.01 ± 0.3a	9.72 ± 0.53b

^†^ *C* = *C*_0_/(1 + *C*_0_ *Kt*), where *C* is the concentration of the phenolic compound (g/100 g extract), *C*_0_ is the initial concentration of the phenolic compound (g/100 g extract), *K* is the reaction rate constant ((g/100 g)^−1^ days^−1^), and *t* is the time in days.

^‡^ 3‐β‐D‐Glucopyranosyl‐4‐*O*‐β‐D‐glucopyranosyliriflophenone.

^§^ 3′,5′‐di‐β‐D‐Glucopyranosyl‐3‐hydroxyphloretin.

^¶^ 3′,5′‐di‐β‐D‐Glucopyranosylphloretin.

* Means with the same letter for a compound/moisture uptake in the two samples (CSE and CSE‐NV) stored at ca 7%, 53%, and 75% RH at 25 and 40°C are not significantly different (*p ≥* 0.05).

A comparison of the phenolic compounds revealed that HPDG had the highest degradation rate constants for all samples and storage conditions (0.811–8.878 ((g/100 g)^−1^ days^−1^) × 10^3^, Table 4). Its reaction rate constants were also significantly higher than that of PDG (0.322–2.071 ((g/100 g)^−1^ days^−1^) × 10^3^), its 3‐deoxy derivative. The reaction rate constant for HPDG could be decreased with phytosomal formulation, but it remained unstable relative to the other phenolic compounds present in the CSE‐NVs. Isomangiferin, which could be ranked next in terms of instability, was less stable than its regio‐isomer, mangiferin, based on its reaction rate constants. For example, at 25°C and 7% RH, isomangiferin had a reaction rate constant of 1.264 ((g/100 g)^−1^ days^−1^) × 10^3^, whereas that of mangiferin was 0.321 ((g/100 g)^−1^ days^−1^) × 10^3^ (Table 4).

**FIGURE 6:**
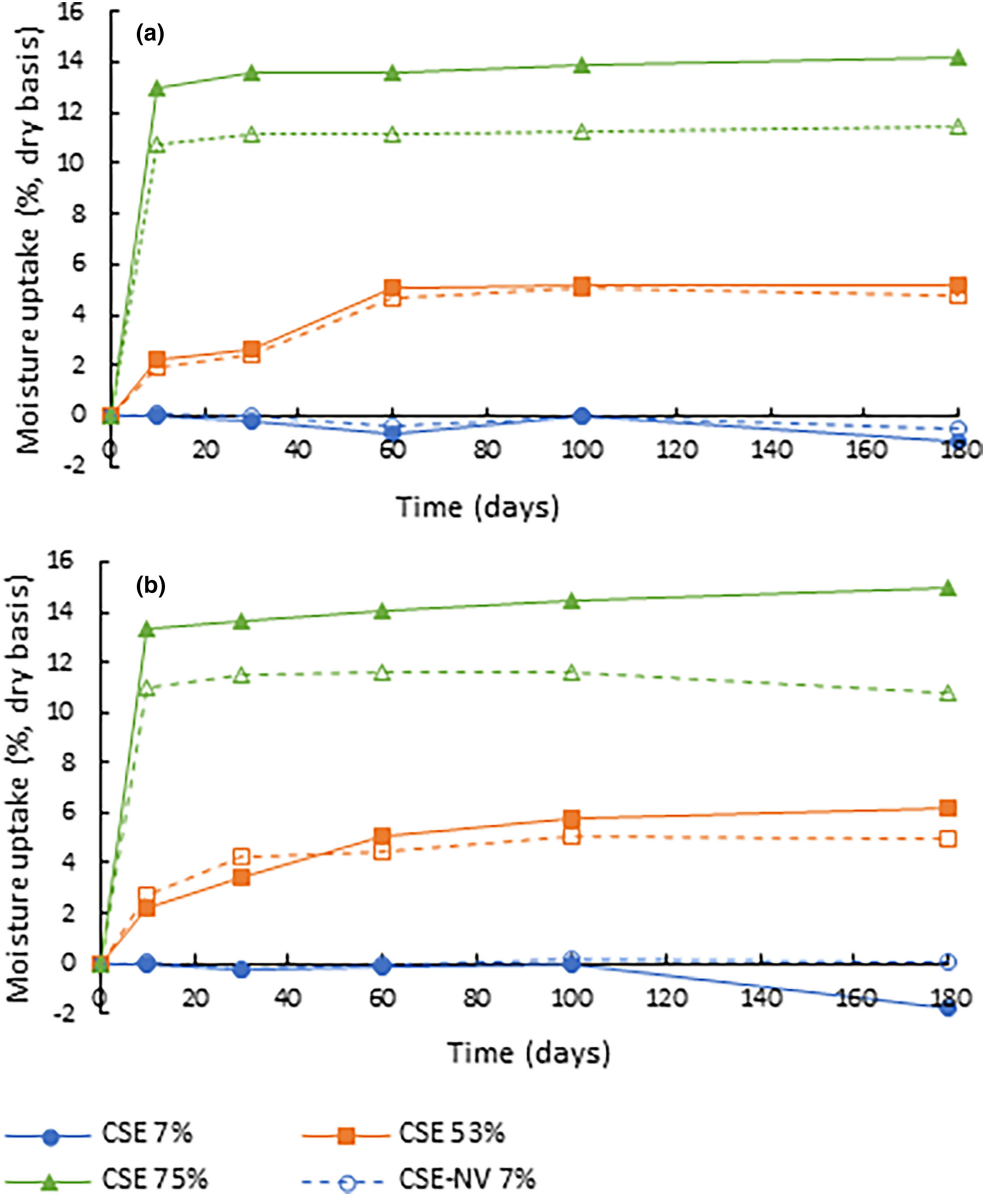
Moisture uptake (%, dry basis) of a *Cyclopia subternata* extract (CSE) and CSE nano‐phytosome vesicles (CSE‐NV) during storage at different temperatures and relative humidity (RH) combinations (7%, 53%, and 75% RH at (a) 25°C and (b) 40°C) for 180 days. Negative values indicate moisture loss.

In most cases, increasing the temperature from 25 to 40°C increased the degradation rate constants of the compounds (Table 4). This effect only becomes predominantly significant (*p* < 0.05) at 75% RH; at 7% RH, the increase in reaction rate constants with an increase in temperature varies between 1.0‐ and 2.5‐fold, at 53% between 1.0‐ and 4.0‐fold, and 75% between 1.2‐ and 5.4‐fold. The reaction rate constants also increased as RH increased. This effect for the individual compounds was only predominantly significant (*p* < 0.05) between 53% and 75% RH, but not between 7% and 53% RH. The reaction rate constants of the compounds ranged between 0.200–1.865, 0.322–5.416, and 0.382–8.878 ((g/100 g)^−1^ days^−1^) × 10^3^ at 7%, 53%, and 75% RH, respectively. The pronounced effect of higher RH can be correlated with an increase in MC of the samples due to markedly higher moisture sorption (Figure 6) and as indicated by the MSI data at higher RH (Figure 5). Note that the negative moisture uptake at 7% RH is the result of a decrease in the mass of the powder during storage due to moisture loss.

Complexation of the compounds by PC affected their reaction rate constants. Scolymoside, HPDG, and PDG had lower reaction rate constants in CSE‐NV compared to CSE. On the other hand, IDG, mangiferin, and isomangiferin showed the opposite trend at higher RH values. However, considering the average phenolic stability of the quantified compounds at 7% RH, phytosomal complexation (CSE‐NV vs. CSE) decreased overall phenolic degradation by 5.5% and 2.6% at 25 and 40°C, respectively (Table 5). At 75% RH, phytosomal complexation decreased phenolic degradation by 1.6% and 5.6% at 25 and 40°C, respectively (Table 5).

**TABLE 5:** Degradation (%, *n* = 3, average ± SD) of a green *Cyclopia subternata* extract (CSE) and green *Cyclopia subternata* extract nano‐phytosome vesicles (CSE‐NV) during storage at different temperature and relative humidity (RH) combinations (7%, 53%, and 75% RH at 25 and 40°C) for 180 days.

Compound	RH (%)	7%		53%		75%	
	Temperature (°C)	CSE	CSE‐NV	CSE	CSE‐NV	CSE	CSE‐NV
IDG^†^	25	12.1 ± 0.2ef*	11.3 ± 3.6fg	12.3 ± 0.9ef	10.7 ± 1.9g	13.4 ± 0.2cd	12.8 ± 2.3de
	40	12.7 ± 0.4de	10.7 ± 4.3g	14.4 ± 0.6c	14 ± 2.9c	18.3 ± 0.5a	16.6 ± 0.8b
HPDG^‡^	25	12.5 ± 1.1g	5.1 ± 1.3h	13.8 ± 1.3fg	13.6 ± 1.6fg	21 ± 0.6e	19.6 ± 1.8e
	40	15.1 ± 0f	15.5 ± 0.5fg	25.2 ± 0.2d	32.1 ± 1.8c	48.4 ± 0.7a	44.2 ± 2.6b
Eriocitrin	25	12.7 ± 1b	7.4 ± 1.2gh	12 ± 1bcd	7.9 ± 2fgh	12.8 ± 1.6b	10 ± 2.4def
	40	12.3 ± 1.1bc	6.3 ± 0.6h	9.4 ± 0.6efg	10.3 ± 0.9cde	18.9 ± 0.3a	11.8 ± 0bcd
PDG^§^	25	12.5 ± 0.3h	7 ± 1.4j	13.6 ± 0.3g	10.9 ± 0.9i	17.7 ± 0.6d	15.6 ± 1.5e
	40	13.9 ± 0.8g	14.8 ± 0.5f	21.2 ± 0.6c	27.3 ± 0.4b	38.1 ± 0.3a	27.7 ± 0.5b
Hesperidin	25	11.5 ± 0.7bcd	5.3 ± 1.6g	11.6 ± 1bcd	8.2 ± 1.6f	13.4 ± 1b	9.1 ± 1.6ef
	40	11 ± 0.8cd	7.9 ± 1.5fg	12.8 ± 0.6bc	10.3 ± 0.4de	30.9 ± 1.9a	10.2 ± 0.7de
Mangiferin	25	12.9 ± 0.1h	7.4 ± 0.9j	13.9 ± 0.6h	16.1 ± 0.7g	31.4 ± 0.8e	39.3 ± 0.7de
	40	13.2 ± 0.7h	10.3 ± 0.9i	20.6 ± 0.6fg	46.6 ± 1c	70.8 ± 0.2b	84.7 ± 0.3a
Isomangiferin	25	12.9 ± 0.2	1.3 ± 1.9	14.7 ± 2.1	7.2 ± 1	17.8 ± 0.8	15.2 ± 2.5
	40	12.8 ± 0.4	6.1 ± 1	16.2 ± 0.5	20.3 ± 0.5	38.5 ± 0.3	35.5 ± 1.8
Vicenin‐2	25	10.7 ± 0.9d	10.1 ± 2.2de	11.1 ± 0.6d	7.3 ± 1g	9.7 ± 0.9def	8.5 ± 2.3fg
	40	10.7 ± 1.5d	9 ± 2.5ef	12.6 ± 0.4c	13.7 ± 1.4bc	14.6 ± 1.7b	16.7 ± 0.6a
Scolymoside	25	16.6 ± 0.4b	9.8 ± 3cd	15.2 ± 0.7b	12.3 ± 1.4c	17.2 ± 1b	10.6 ± 3.7cd
	40	15.7 ± 0.8b	12.3 ± 0.5c	16.9 ± 0.7b	11.1 ± 0.5cd	28.7 ± 1.2a	9 ± 3.1d
Extent of degradation of the sum of quantified compounds	25	12.7	7.2	13.1	10.5	17.2	15.6
	40	12.9	10.3	16.6	20.6	34.1	28.5

^†^ 3‐β‐D‐Glucopyranosyl‐4‐*O*‐β‐D–glucopyranosyliriflophenone.

^‡^ 3′,5′‐di‐β‐D‐Glucopyranosyl‐3‐hydroxyphloretin.

^§^ 3′,5′‐di‐β‐D‐Glucopyranosylphloretin.

* Means with the same letter for a compound in the two samples (CSE and CSE‐NV) stored at ca 7, 53, and 75% RH at 25 and 40°C are not significantly different (*p ≥* 0.05).

## 4 DISCUSSION

### 4.1 Formulation and production of CSE‐NV

The ingredients were selected based on the requirements for creating a food‐grade, more cost‐effective functional food ingredient. CSE was chosen as an interesting example of a bioactive extract (Yoshida et al., 2020) with relatively high levels of compounds belonging to different phenolic classes, for example, xanthone, benzophenone, dihydrochalcone, flavone, and flavanone. The major phenolic compounds present in CSE were the following (g/100 g extract): IDG (2.31); IMG (0.70); HPDG (0.36); PDG (1.35), hesperidin (0.99), mangiferin (2.41), isomangiferin (0.62), vicenin‐2 (0.20), and scolymoside (1.14). The poor stability of several of these phenolic compounds in a spray‐dried powder during shelf‐life storage (De Beer et al., 2018) and a model ready‐to‐drink beverage (Human et al., 2021) motivated the preparation of phytosomes. PC was selected as an unrefined, cost‐effective source of phosphatidylcholine for complex formation. Phosphatidylcholine also has anti‐inflammatory properties (Treede et al., 2007), providing further justification for its use.

### 4.2 Effect of formulation and process parameters on physical properties of CSE‐NV

Nano‐phytosome behavior during storage is strongly dependent on the physical properties of the phytosome such as its composition and its vesicle shape and size. This study showed that the process parameters have a significant and controllable effect on these vesicle properties.

#### 4.2.1 Vesicle size, PDI, and zeta potential

Smaller vesicle size and PDI have been linked to better emulsion stability in solution (Molaveisi et al., 2021; Saari & Chua, 2020) and increased in vitro circulation, dissolution, and absorption (McClements & Öztürk, 2021). The results showed that the vesicle size can be controlled by the sonication time during phytosome production. The initial decrease in vesicle size with increased sonication time of up to 30 min can be attributed to the input of energy that breaks down the vesicles into nano‐size droplets due to an increase in the external pressure (Das et al., 2011). The subsequent increase in vesicle size with sonication longer than 35 min is likely due to “over‐processing” and vesicle coalescence (Desrumaux & Marcand, 2002; Tornberg, 1980). Choosing an appropriate sonication time is thus critical, considering the application; for example, for oral delivery, the optimal vesicle size is between 50 and 200 nm (Chan et al., 2023).

The PDI, a measure of the uniformity of vesicle size, was increased by an increase in the sonication time. This increase in PDI can be attributed to vesicle coalescence (Desrumaux & Marcand, 2002). Even though a lower PDI is preferable, the PDI values obtained could be classified collectively as being “nearly monodisperse” (Stetefeld et al., 2016) and the increase in PDI was thus not deemed critical.

The zeta potential is a measure of the surface charge potential of the vesicles, with a higher absolute value indicating greater repulsion between vesicles and theoretically higher solution stability (Barani et al., 2021). It is widely assumed that an absolute zeta potential greater than 30 mV confers stability on a nano‐system (Wang et al., 2015), which was the case for the nano‐phytosomes produced at all CCD conditions in this study.

#### 4.2.2 Yield, EE, and LC

Like the reaction yield, the EE is also a measure of the efficiency of the process because the unbound extract is considered waste. The LC will ultimately determine the product dose of the nanovesicles, since a higher LC may result in a lower dose, which is preferable due to cost and biological implications. The yield was not affected by any of the process parameters that were varied. This is likely because the yield is a function of good synthesis practices that ensure the quantitative transfer of the reaction medium between production steps. The EE and LC, based on the $\sum_{\mathrm{QP}}$, could be solely controlled by the PC:CSE ratio. This PC:CSE ratio is assumed to translate to the stoichiometric mass ratio at which the OH groups from the phenolic mixture of the extract bind to the phosphate groups of phosphatidylcholine. The EE of the individual phenolic compounds differed (Table 2). Permana et al. (2020) suggested that the ability of a compound to bind to phosphatidylcholine is linked to its number of hydrogen bond acceptors and donors, where more hydrogen bond acceptors and donors result in higher binding efficiency. However, this was not observed in the current study, considering IMG and IDG. Irrespective of the process conditions, IMG and IDG showed the highest and lowest binding capacity for phosphatidylcholine in PC, respectively (Table 2), despite IMG having fewer hydrogen bond acceptors and donors than IDG (Miller et al., 2021). This implies that other chemical reaction‐related factors, such as steric hindrance, solubility, and/or hydrophobicity, may also play a role during food‐grade phytosome formation.

### 4.3 DSC, FTIR XRPD, and NMR of CSE‐NV—Confirmation of hydrogen bonding

The thermal behavior, chemical nature, and crystal structure of the individual constituents, as well as CSE‐NV, were investigated to confirm the binding of the phenolic compounds with phosphatidylcholine. The appearance of a small group of endothermic peaks between 140 and 150°C in the DSC thermogram of CSE‐NV, while absent in the DSC thermograms of the individual components of the phytosome (PC and CSE), is an indication that complexation via hydrogen bonding of the phospholipid and phenolic compounds took place (Babazadeh et al., 2019).

A marked decrease in the crystallinity observed for phytosomes compared to their components can also be considered an indication of hydrogen bonding between a phenolic compound and a phospholipid (Babazadeh et al., 2019). In the current study, PC and CSE were already largely amorphous, complicating this interpretation of the XRPD diffractograms because the changes observed in the CSE‐NV diffractogram were very subtle. However, the disappearance of the broad halo peak in the CSE and PC diffractograms, as well as the disappearance of the small peak at 2θ = 38.2° present in the CSE diffractogram, indicates a change in the crystal structure of the ingredients and formation of a phytosome complex.

FTIR provides confirmation of hydrogen bond formation and information on the chemical groups that are involved in the bonding (Chi et al., 2020). The complexation of the free hydroxyl groups of the phenolic compounds with the polar choline section of PC was indicated by a slight enlargement of the O–H band and deformation of the P=O stretching band annotated in the FTIR spectra (Figure 4), respectively. The similarity of the carboxyl moiety band in terms of shape and size in the FTIR spectra of both CSE and CSE‐NV implies that this functionality is not involved in the formation of the phytosome complex (Chi et al., 2020).

The suppression and broadening of the NMR signals corresponding to the N–(CH_3_)_3_ (at ca 3.2–3.4 ppm) and CH_2_‐P‐O (between 4–6 ppm) groups in CSE‐NV, compared to PC, indicate that these groups are involved in hydrogen bonding (El‐Gazayerly et al., 2014). Furthermore, typically, phenol functional groups and some carbonyl groups, which are abundant in the various phenolic compounds of CSE, will give rise to signals between 4–7 and 9–10 ppm, respectively (Pavia et al., 2009). Contrary to the FTIR data, the suppression of the signals for the carbonyl groups in CSE‐NV confirms that this functional group of the phenolic compounds are involved in the complex formation with PC.

### 4.4 Stability of CSE‐NV in powder form

#### 4.4.1 MSI

Before the stability trial, the thermodynamic link between the environmental RH and equilibrium MC of CSE, PC, and CSE‐NV was determined, using their MSIs. Their respective MSIs could indicate their behavior toward moisture during the stability trial. The smaller hysteresis loop of CSE‐NV compared to CSE indicated that the complexation of CSE with PC will likely affect the stability of CSE positively. Hysteresis occurs when physical changes in the powders expose new sites that can adsorb moisture, and a large hysteresis loop indicates that exposure of the powder to moisture will lead to long‐lasting physical changes in the powder, as well as compromise its stability (Al‐Muhtaseb et al., 2002).

The M_0_ values provide an indication of water molecules strongly bound to hydrophilic surface sites. For a shelf‐stable product, its MC must be less than the predicted M_0_ (Labuza & Altunakar, 2007). This was true for PC and CSE‐NV but not for CSE (Table 3), a further indication that complexing CSE with PC may offer CSE protection against changes in environmental humidity.

#### 4.4.2 Stability trial

The effects of temperature (25 and 40°C) and RH (7%, 53%, and 75%) on the stability of CSE‐NV in dry form were studied over 180 days (ca 6 months). Storage temperatures were chosen per ICH guidelines (ICH Q1A(R2), 2003). These temperatures and RH values were also considered realistic for processing, transport, and storage of a dry product in situations when environmental conditions are not controlled.

The poor stability of HPDG, as highlighted by its high reaction rate constants, was not unexpected given its poor stability observed when CSE was used to formulate ready‐to‐drink model beverage solutions (Human et al., 2021). The poor stability of HPDG is attributed to the ortho‐dihydroxy group on its B‐ring and its involvement in oxidation in various sample matrices as reviewed by De Beer et al. (2022). The higher instability of isomangiferin compared to mangiferin was similar to what was found in heat preservation experiments of CSE in solution (Human et al., 2021). However, during storage of a spray‐dried aqueous *C. subternata* extract powder at 25 and 40°C/65% RH, mangiferin was less stable than isomangiferin (De Beer et al., 2018). This discrepancy in the stability of these two compounds could likely be due to differences in the interconversion rate between mangiferin and isomangiferin (Beelders et al., 2017), depending on the degradation conditions and extract matrix.

As expected, compound stability in the CSE and CSE‐NV powders decreased with an increase in storage temperature and RH. In the presence of moisture and at higher temperatures, more energy is available for molecular motion, allowing more interaction between constituents, which in turn would accelerate compound degradation (Mauer & Taylor, 2010).

Comparison of CSE and CSE‐NV to assess the potential stabilizing effect of phytosomal formulation becomes more complicated because the phenolic degradation in these samples appears to be dependent on the compound structure and storage condition, rather than following a general trend. Despite this caveat, the average phenolic stability of the quantified compounds in CSE and CSE‐NV at each condition showed that phytosomal formulation of CSE could offer better protection during storage at lower RH values, whereas at higher RH values, the effect of the increase in MC outweighs the effect of phytosomal formulation for selected phenolic compounds.

Future research should investigate the difference in the affinity of the phenolic compounds to bind and remain bound to phosphatidylcholine in the presence of excess moisture due to a high RH environment.

## 5 CONCLUSIONS

Food‐grade, cost‐effective nano‐phytosomes for phenolic compounds in a green *C. subternata* extract were successfully prepared. The results provide a general indication of factors that are important in food‐grade phytosomal formulation. These results can be used as a starting point to produce food‐grade phytosomes based on a variety of polar phenolic extracts. However, the study is limited by the number of process parameters investigated. The complexation of the phenolic compounds present in the *C. subternata* extract can protect selected compounds during the storage of the dried phytosomes. However, low relative humidity and storage temperature would still be required. Future research should concentrate on the challenges of incorporating the phytosomes into a functional food formulation such as a powdered iced tea or ready‐to‐drink iced tea formulation with a standardized amount of bioactive phenolic compounds. This will open new challenges such as possible interaction of other food ingredients with the nano‐phytosomes, sensory profile, and appearance of the food product.

## AUTHOR CONTRIBUTIONS

**Chantelle Human:** Conceptualization (lead); funding acquisition (lead); investigation (lead); project administration (lead); visualization (lead); writing – original draft (lead). **Marique Aucamp:** Investigation (lead); resources (lead). **Dalene de Beer:** Conceptualization (supporting); resources (lead); writing – review and editing (equal). **Marieta van der Rijst:** Formal analysis (supporting). **Elizabeth Joubert:** Conceptualization (supporting); resources (lead); writing – review and editing (lead).

## CONFLICT OF INTEREST STATEMENT

All authors declare that they have no conflicts of interest.

## ETHICS STATEMENT

This study does not involve any human or animal testing.

## Supporting information


Data S1.

## Data Availability

The data that support the findings of this study are available on request from the corresponding author.
