# Supplementary material for: Food‐grade phytosome vesicles for nanoencapsulation of labile C‐glucosylated xanthones and dihydrochalcones present in a plant extract matrix—Effect of process conditions and stability assessment
Source: Food Sci Nutr. 2023 Oct 6;11(12):8093–111. doi: 10.1002/fsn3.3730 (PMC10724603; doi:10.1002/fsn3.3730)
Supplement: Supplementary file 1 — Data S1. [file FSN3-11-8093-s001.docx]

**Supplementary Material**

**Food-grade phytosome vesicles for nanoencapsulation of labile *C*-glucosylated xanthones and dihydrochalcones present in a plant extract matrix – effect of process conditions and stability assessment**

**Short running title:** **Formulation of food-grade phytosomes**

Chantelle Human^1,*^ | Marique Aucamp^2^ | Dalene de Beer^1,3^ | Marieta van der Rijst^4^ | Elizabeth Joubert^1,3^

^1^ Plant Bioactives Group, Post-Harvest and Agro-Processing Technologies, Agricultural Research Council (Infruitec-Nietvoorbij), Private Bag X5026, Stellenbosch 7599, South Africa

^2^ School of Pharmacy, University of the Western Cape, Private Bag X17, Bellville 7535, South Africa;

^3^ Department of Food Science, Stellenbosch University, Private Bag X1, Matieland (Stellenbosch) 7600, South Africa

^4^ Biometry Unit, Agricultural Research Council, Private Bag X5026, Stellenbosch 7599, South Africa.

**^*^ Correspondence:** HumanC@arc.agric.za; Tel.: (021)-809-341

**TABLE S.1** The coefficient of determination (R^2^) for the Guggenheim–Anderson de Boer (GAB) and Brunauer–

| Sample | GAB | BET |
| --- | --- | --- |
| CSE | 0.9054 | 0.9327 |
| PC | 0.7931 | 0.5939 |
| CSE-NV | 0.7804 | 0.5074 |
| ^†^*Cyclopia subternata* extract; ^‡^lipoid S 40 (fat-free soybean lecithin containing 40% phosphatidylcholine); ^§^*Cyclopia subternata* extract nano-phytosome vesicles. | | |

Emmet–Teller (BET) models fitted to moisture adsorption data obtained at 25 °C for CSE^†^, PC^‡^ and CSE-NV^§^.

**TABLE S.2** Saturated salt solutions used to create fixed relative humidity (RH) conditions of ca 7, 53 and 75% based on Greenspan (1977) for storage of CSE^†^ and CSE-NV^‡^ at 25 and 40 °C for 180 days.

| RH condition (%) | Temperature (°C) | Salt | Salt concentration (mg/mL) |
| --- | --- | --- | --- |
| 7 (8.24 ± 0.21) ^§^ | 25 | potassium hydroxide (KOH) | 0.85 |
| 7 (6.26 ± 0.17) | 40 | potassium hydroxide (KOH) | 0.85 |
| 53 (52.89 ± 0.22) | 25 | magnesium nitrate (Mg(NO_3_)_2_) | 0.70 |
| 53 (53.17 ± 0.41) | 40 | sodium bromide (NaBr) | 1.00 |
| 75 (75.29 ± 0.12) | 25 | sodium chloride (NaCl) | 0.36 |
| 75 (74.68 ± 0.13) | 40 | sodium chloride (NaCl) | 0.36 |
| ^†^*Cyclopia subternata* extract; ^‡^*Cyclopia subternata* extract nano-phytosome vesicles; ^§^Exact values as given by Greenspan (1977). | | | |

| Parameter | Regression Coefficients | Sum of squares | Degrees of freedom | Mean square | F | P |
| --- | --- | --- | --- | --- | --- | --- |
| Yield (%) |  |  |  |  |  |  |
| Intercept | 94.74143 |  |  |  |  |  |
| (1) Temperature (°C) (L)^§§^ | 0.01968 | 1.356439 | 1 | 1.356439 | 3.477511 | 0.121219 |
| Temperature (°C) (Q)^¶¶^ | -0.00091 | 0.230358 | 1 | 0.230358 | 0.590569 | 0.476908 |
| (2) Sonication time (min) (L) | 0.00548 | 0.344160 | 1 | 0.344160 | 0.882325 | 0.390691 |
| Sonication time (min)(Q) | -0.00024 | 0.082349 | 1 | 0.082349 | 0.211118 | 0.665178 |
| (3) PC:CSE (m/m) (L) | 0.09107 | 0.630372 | 1 | 0.630372 | 1.616089 | 0.259565 |
| PC:CSE (m/m) (Q) | -0.05714 | 1.077005 | 1 | 1.077005 | 2.761124 | 0.157470 |
| 1L by 2L | -0.00004 | 0.000703 | 1 | 0.000703 | 0.001803 | 0.967774 |
| 1L by 3L | 0.01016 | 0.328727 | 1 | 0.328727 | 0.842760 | 0.400725 |
| 2L by 3L | 0.00054 | 0.002085 | 1 | 0.002085 | 0.005346 | 0.944547 |
| Lack of Fit |  | 3.121602 | 5 | 0.624320 | 1.600574 | 0.309158 |
| Pure Error |  | 1.950302 | 5 | 0.390060 |  |  |
| Total SS |  | 8.608601 | 19 |  |  |  |
| R^2^ |  |  |  |  |  | 0.41083 |
| R^2^_adj_ |  |  |  |  |  | 0.39006 |
| Vesicle size (nm) |  |  |  |  |  |  |
| Intercept | 122.1949 |  |  |  |  |  |
| (1) Temperature (°C) (L) | 7.1044 | 11.77 | 1 | 11.766 | 0.3836 | 0.562830 |
| Temperature (°C) (Q) | -0.0481 | 646.06 | 1 | 646.056 | 21.0606 | 0.005898 |
| (2) Sonication time (min) (L) | -3.8593 | 3550.60 | 1 | 3550.595 | 115.7451 | 0.000120 |
| Sonication time (min)(Q) | 0.0807 | 9205.76 | 1 | 9205.757 | 300.0965 | 0.000012 |
| (3) PC:CSE (m/m) (L) | -28.1904 | 5929.60 | 1 | 5929.600 | 193.2978 | 0.000035 |
| PC:CSE (m/m) (Q) | 2.1904 | 1582.52 | 1 | 1582.523 | 51.5883 | 0.000814 |
| 1L by 2L | -0.0654 | 1537.81 | 1 | 1537.813 | 50.1309 | 0.000870 |
| 1L by 3L | -0.2498 | 198.72 | 1 | 198.723 | 6.4781 | 0.051565 |
| 2L by 3L | 0.2902 | 603.60 | 1 | 603.597 | 19.6765 | 0.006791 |
| Lack of Fit |  | 12993.10 | 5 | 2598.620 | 84.7119 | 0.000079 |
| Pure Error |  | 153.38 | 5 | 30.676 |  |  |
| Total SS |  | 36683.33 | 19 |  |  |  |
| R^2^ |  |  |  |  |  | 0.641620 |
| R^2^_adj_ |  |  |  |  |  | 0.319080 |

**TABLE S.3** ANOVA results of polynomial regression for the response factors (vesicle size, PDI^†^, zeta potential, yield, EE^‡^ and LC^§^) based on the process parameters (temperature (°C), sonication time (min) and PC^¶^:CSE^††^ ratio (% m/m)) of a central composite design to produce CSE-NV^‡‡^*.*

| **TABLE S.3** continued | | | | | | |
| --- | --- | --- | --- | --- | --- | --- |
| Parameter | Regression Coefficients | Sum of squares | Degrees of freedom | Mean square | F | P |
| PDI |  |  |  |  |  |  |
| Intercept | 0.644888 |  |  |  |  |  |
| (1) Temperature (°C) (L) | -0.008696 | 0.000231 | 1 | 0.000231 | 0.16642 | 0.700195 |
| Temperature (°C) (Q) | 0.000100 | 0.002768 | 1 | 0.002768 | 1.99528 | 0.216900 |
| (2) Sonication time (min) (L) | -0.004839 | 0.013583 | 1 | 0.013583 | 9.78954 | 0.025992 |
| Sonication time (min)(Q) | 0.000106 | 0.015892 | 1 | 0.015892 | 11.45407 | 0.019580 |
| (3) PC:CSE (m/m) (L) | -0.038057 | 0.002994 | 1 | 0.002994 | 2.15774 | 0.201793 |
| PC:CSE (m/m) (Q) | 0.001358 | 0.000608 | 1 | 0.000608 | 0.43817 | 0.537290 |
| 1L by 2L | -0.000059 | 0.001250 | 1 | 0.001250 | 0.90093 | 0.386120 |
| 1L by 3L | 0.000322 | 0.000330 | 1 | 0.000330 | 0.23750 | 0.646634 |
| 2L by 3L | 0.000818 | 0.004790 | 1 | 0.004790 | 3.45241 | 0.122275 |
| Lack of Fit |  | 0.007723 | 5 | 0.001545 | 1.11325 | 0.454576 |
| Pure Error |  | 0.006937 | 5 | 0.001387 |  |  |
| Total SS |  | 0.059636 | 19 |  |  |  |
| R^2^ |  |  |  |  |  | 0.754170 |
| R^2^_adj_ |  |  |  |  |  | 0.532920 |
| Zeta potential (mV) |  |  |  |  |  |  |
| Intercept | -46.8282 |  |  |  |  |  |
| (1) Temperature (°C) (L) | -0.1299 | 40.1627 | 1 | 40.16268 | 22.75627 | 0.005013 |
| Temperature (°C) (Q) | 0.0013 | 0.5087 | 1 | 0.50870 | 0.28823 | 0.614375 |
| (2) Sonication time (min) (L) | 0.5265 | 29.8018 | 1 | 29.80183 | 16.88579 | 0.009271 |
| Sonication time (min)(Q) | -0.0065 | 58.9475 | 1 | 58.94748 | 33.39978 | 0.002182 |
| (3) PC:CSE (m/m) (L) | -1.7153 | 6.6350 | 1 | 6.63497 | 3.75939 | 0.110224 |
| PC:CSE (m/m) (Q) | -0.0444 | 0.6514 | 1 | 0.65138 | 0.36907 | 0.570041 |
| 1L by 2L | -0.0001 | 0.0011 | 1 | 0.00114 | 0.00065 | 0.980696 |
| 1L by 3L | 0.0438 | 6.1006 | 1 | 6.10058 | 3.45660 | 0.122097 |
| 2L by 3L | -0.0146 | 1.5307 | 1 | 1.53066 | 0.86727 | 0.394457 |
| Lack of Fit |  | 41.6223 | 5 | 8.32446 | 4.71666 | 0.056949 |
| Pure Error |  | 8.8245 | 5 | 1.76491 |  |  |
| Total SS |  | 208.2898 | 19 |  |  |  |
| R^2^ |  |  |  |  |  | 0.757800 |
| R^2^_adj_ |  |  |  |  |  | 0.539830 |
| EE (%) |  |  |  |  |  |  |
| Intercept | -48.0741 |  |  |  |  |  |
| (1) Temperature (°C) (L) | -0.0746 | 2.33 | 1 | 2.325 | 0.1803 | 0.688731 |
| Temperature (°C) (Q) | 0.0022 | 1.35 | 1 | 1.347 | 0.1045 | 0.759609 |
| (2) Sonication time (min) (L) | 0.0405 | 29.50 | 1 | 29.501 | 2.2883 | 0.190762 |
| Sonication time (min)(Q) | -0.0001 | 0.03 | 1 | 0.027 | 0.0021 | 0.965486 |
| (3) PC:CSE (m/m) (L) | 39.5095 | 9288.08 | 1 | 9288.079 | 720.4445 | 0.000001 |
| PC:CSE (m/m) (Q) | -2.9157 | 2804.11 | 1 | 2804.109 | 217.5052 | 0.000026 |
| 1L by 2L | -0.0011 | 0.47 | 1 | 0.472 | 0.0366 | 0.855728 |
| 1L by 3L | -0.0149 | 0.71 | 1 | 0.706 | 0.0548 | 0.824210 |
| 2L by 3L | 0.0288 | 5.93 | 1 | 5.933 | 0.4602 | 0.527663 |
| Lack of Fit |  | 1104.02 | 5 | 220.803 | 17.1270 | 0.003656 |
| Pure Error |  | 64.46 | 5 | 12.892 |  |  |
| Total SS |  | 11285.75 | 19 |  |  |  |
| R^2^ |  |  |  |  |  | 0.896460 |
| R^2^_adj_ |  |  |  |  |  | 0.803280 |

| Parameter | Regression Coefficients | Sum of squares | Degrees of freedom | Mean square | F | P |
| --- | --- | --- | --- | --- | --- | --- |
| LC (%) |  |  |  |  |  |  |
| Intercept | -8.74781 |  |  |  |  |  |
| (1) Temperature (°C) (L) | 0.07645 | 0.1296 | 1 | 0.1296 | 0.1653 | 0.701125 |
| Temperature (°C) (Q) | -0.00061 | 0.1038 | 1 | 0.1038 | 0.1325 | 0.730749 |
| (2) Sonication time (min) (L) | 0.02463 | 1.1643 | 1 | 1.1643 | 1.4857 | 0.277255 |
| Sonication time (min)(Q) | -0.00057 | 0.4572 | 1 | 0.4572 | 0.5834 | 0.479471 |
| (3) PC:CSE (m/m) (L) | 7.66705 | 177.5593 | 1 | 177.5593 | 226.5659 | 0.000023 |
| PC:CSE (m/m) (Q) | -0.69727 | 160.3638 | 1 | 160.3638 | 204.6244 | 0.000030 |
| 1L by 2L | 0.00016 | 0.0095 | 1 | 0.0095 | 0.0121 | 0.916771 |
| 1L by 3L | -0.00505 | 0.0813 | 1 | 0.0813 | 0.1038 | 0.760378 |
| 2L by 3L | 0.00526 | 0.1982 | 1 | 0.1982 | 0.2528 | 0.636437 |
| Lack of Fit |  | 125.8972 | 5 | 25.1794 | 32.1290 | 0.000832 |
| Pure Error |  | 3.9185 | 5 | 0.7837 |  |  |
| Total SS |  | 331.2594 | 19 |  |  |  |
| R^2^ |  |  |  |  |  | 0.608110 |
| R^2^_adj_ |  |  |  |  |  | 0.255420 |
|  |  |  |  |  |  |  |
| ^†^Polydispersity index; ^‡^encapsulation efficiency; ^§^loading capacity; ^¶^lipoid S 40 (fat-free soybean lecithin containing 40% phosphatidylcholine); ^††^*Cyclopia subternata* extract; ^‡‡^*Cyclopia subternata* extract nano-phytosome vesicles; ^§§^linear coefficient; ^¶¶^quadratic coefficient. | | | | | | |

**TABLE S.3** continued

| Sample | Temperature (°C) | RH (%) | Compound | R^2^_adj_ | | | | | | |
| --- | --- | --- | --- | --- | --- | --- | --- | --- | --- | --- |
|  |  |  |  | Zero-order | | First-order | | Second-order | | Fractional |
| CSE | 25 | 7 | IDG^¶¶^ | 0.9732 | | 0.9717 | | 0.9689 | | 0.9639 |
| CSE | 25 | 53 |  | 0.9110 | | 0.9153 | | 0.9187 | | 0.9008 |
| CSE | 25 | 75 |  | 0.8193 | | 0.8357 | | 0.8516 | | 0.9344 |
| CSE | 40 | 7 |  | 0.8713 | | 0.8803 | | 0.8885 | | 0.8995 |
| CSE | 40 | 53 |  | 0.7531 | | 0.7743 | | 0.7954 | | 0.9572 |
| CSE | 40 | 75 |  | 0.9175 | | 0.9167 | | 0.9148 | | 0.8905 |
| CSE-NV | 25 | 7 |  | 0.5505 | | 0.5577 | | 0.5645 | | 0.4746 |
| CSE-NV | 25 | 53 |  | 0.6651 | | 0.6840 | | 0.7032 | | 0.8807 |
| CSE-NV | 25 | 75 |  | 0.7604 | | 0.7733 | | 0.7862 | | 0.8628 |
| CSE-NV | 40 | 7 |  | 0.5899 | | 0.6038 | | 0.6178 | | 0.7255 |
| CSE-NV | 40 | 53 |  | 0.7013 | | 0.7163 | | 0.7291 | | 0.7002 |
| CSE-NV | 40 | 75 |  | 0.7705 | | 0.7787 | | 0.7851 | | 0.7299 |
|  |  |  |  |  | |  | |  | |  |
| CSE | 25 | 7 | HPDG^†††^ | 0.8400 | | 0.8289 | | 0.8172 | | 0.7836 |
| CSE | 25 | 53 |  | 0.7940 | | 0.7863 | | 0.7775 | | 0.7251 |
| CSE | 25 | 75 |  | 0.9448 | | 0.9402 | | 0.9326 | | 0.9258 |
| CSE | 40 | 7 |  | 0.9337 | | 0.9249 | | 0.9148 | | 0.9097 |
| CSE | 40 | 53 |  | 0.9453 | | 0.9464 | | 0.9437 | | 0.9326 |
| CSE | 40 | 75 |  | 0.9729 | | 0.9745 | | 0.9590 | | 0.9688 |
| CSE-NV | 25 | 7 |  | 0.6044 | | 0.6065 | | 0.6084 | | 0.4766 |
| CSE-NV | 25 | 53 |  | 0.8637 | | 0.8623 | | 0.8595 | | 0.8496 |
| CSE-NV | 25 | 75 |  | 0.9239 | | 0.9186 | | 0.9106 | | 0.8995 |
| CSE-NV | 40 | 7 |  | 0.7297 | | 0.7343 | | 0.7386 | | 0.7343 |
| CSE-NV | 40 | 53 |  | 0.9773 | | 0.9747 | | 0.9641 | | 0.9716 |
| CSE-NV | 40 | 75 |  | 0.9659 | | 0.9649 | | 0.9506 | | 0.9573 |
|  |  |  |  |  | |  | |  | |  |
| CSE | 25 | 7 | PDG^‡‡‡^ | 0.7972 | | 0.7876 | | 0.7776 | | 0.7293 |
| CSE | 25 | 53 |  | 0.7864 | | 0.7824 | | 0.7777 | | 0.7149 |
| CSE | 25 | 75 |  | 0.8785 | | 0.8758 | | 0.8723 | | 0.8378 |
| CSE | 40 | 8 |  | 0.8863 | | 0.8789 | | 0.8709 | | 0.8481 |
| CSE | 40 | 53 |  | 0.9018 | | 0.8914 | | 0.8798 | | 0.8688 |
| CSE | 40 | 75 |  | 0.9566 | | 0.9514 | | 0.9374 | | 0.9427 |
| CSE-NV | 25 | 7 |  | 0.8866 | | 0.8839 | | 0.8811 | | 0.8485 |
| CSE-NV | 25 | 53 |  | 0.7540 | | 0.7499 | | 0.7455 | | 0.6725 |
| CSE-NV | 25 | 75 |  | 0.7504 | | 0.7399 | | 0.7293 | | 0.6667 |
| CSE-NV | 40 | 7 |  | 0.7290 | | 0.7175 | | 0.7061 | | 0.6380 |
| CSE-NV | 40 | 53 |  | 0.9242 | | 0.9042 | | 0.8821 | | 0.8987 |
| CSE-NV | 40 | 75 |  | 0.8673 | | 0.8406 | | 0.8125 | | 0.8222 |
|  |  |  |  |  |  | |  | |  | |

**TABLE S.4** R^2^_adj_^†^ between average measured and estimated phenolic compound content (g/100 g extract, n = 3) of CSE^‡^ and CSE-NV^§^ during storage at different temperature and relative humidity (RH) combinations (7%, 53% and 75% RH at 25°C and 40°C) for 180 days. Data were fitted to zero-order^¶^, first-order^††^, second-order^‡‡^ and fractional conversion^§§^ (based on first-order) models.

| **TABLE S.4** Continued | | | | | | | | | | |
| --- | --- | --- | --- | --- | --- | --- | --- | --- | --- | --- |
| Sample | Temperature  (° C) | RH (%) | Compound | R^2^_adj_ | | | | | | |
|  |  |  |  | Zero-order | First-order | Second-order | | | Fractional conversion conversion | |
| CSE | 25 | 7 | Eriocitrin | 0.9466 | 0.9428 | 0.9380 | | | 0.9278 | |
| CSE | 25 | 53 |  | 0.7557 | 0.7535 | 0.7508 | | | 0.6862 | |
| CSE | 25 | 75 |  | 0.8520 | 0.8585 | 0.8644 | | | 0.8557 | |
| CSE | 40 | 7 |  | 0.9135 | 0.9129 | 0.9113 | | | 0.8856 | |
| CSE | 40 | 53 |  | 0.6350 | 0.6460 | 0.6566 | | | 0.5079 | |
| CSE | 40 | 75 |  | 0.7790 | 0.7876 | 0.7959 | | | 0.7750 | |
| CSE-NV | 25 | 7 |  | 0.8404 | 0.8411 | 0.8414 | | | 0.8020 | |
| CSE-NV | 25 | 53 |  | 0.6309 | 0.6338 | 0.6364 | | | 0.5101 | |
| CSE-NV | 25 | 75 |  | 0.9004 | 0.8955 | 0.8900 | | | 0.8694 | |
| CSE-NV | 40 | 7 |  | 0.5769 | 0.5759 | 0.5749 | | | 0.4323 | |
| CSE-NV | 40 | 53 |  | 0.8005 | 0.7901 | 0.7793 | | | 0.7318 | |
| CSE-NV | 40 | 75 |  | 0.8728 | 0.8598 | 0.8460 | | | 0.8272 | |
|  |  |  |  |  | | |  |  | |  |
| CSE | 25 | 7 | Hesperidin | 0.8933 | 0.9002 | 0.9065 | | | 0.9073 | |
| CSE | 25 | 53 |  | 0.6741 | 0.6863 | 0.6983 | | | 0.7260 | |
| CSE | 25 | 75 |  | 0.6361 | 0.6609 | 0.6862 | | | 0.9496 | |
| CSE | 40 | 7 |  | 0.8623 | 0.8680 | 0.8733 | | | 0.8678 | |
| CSE | 40 | 53 |  | 0.8161 | 0.8289 | 0.8413 | | | 0.9214 | |
| CSE | 40 | 75 |  | 0.9671 | 0.9816 | 0.9878 | | | 0.9874 | |
| CSE-NV | 25 | 7 |  | 0.5597 | 0.5686 | 0.5777 | | | 0.8652 | |
| CSE-NV | 25 | 53 |  | 0.4549 | 0.4684 | 0.4822 | | | 0.7285 | |
| CSE-NV | 25 | 75 |  | 0.5618 | 0.5785 | 0.5953 | | | 0.8522 | |
| CSE-NV | 40 | 7 |  | 0.8226 | 0.8272 | 0.8317 | | | 0.8283 | |
| CSE-NV | 40 | 53 |  | 0.9217 | 0.9233 | 0.9245 | | | 0.9019 | |
| CSE-NV | 40 | 75 |  | 0.8683 | 0.8746 | 0.8806 | | | 0.8912 | |
|  |  |  |  |  |  |  | | |  | |
| CSE | 25 | 7 | Mangiferin | 0.9527 | 0.9540 | 0.9540 | | | 0.9393 | |
| CSE | 25 | 53 |  | 0.8759 | 0.8830 | 0.8890 | | | 0.8778 | |
| CSE | 25 | 75 |  | 0.9466 | 0.9671 | 0.9801 | | | 0.9786 | |
| CSE | 40 | 8 |  | 0.9335 | 0.9408 | 0.9470 | | | 0.9490 | |
| CSE | 40 | 53 |  | 0.8974 | 0.9154 | 0.9314 | | | 0.9635 | |
| CSE | 40 | 75 |  | 0.8130 | 0.9558 | 0.9986 | | | 0.9937 | |
| CSE-NV | 25 | 7 |  | 0.9431 | 0.9456 | 0.9477 | | | 0.9539 | |
| CSE-NV | 25 | 53 |  | 0.9172 | 0.9253 | 0.9319 | | | 0.9278 | |
| CSE-NV | 25 | 75 |  | 0.9822 | 0.9894 | 0.9802 | | | 0.9884 | |
| CSE-NV | 40 | 7 |  | 0.6617 | 0.6690 | 0.6762 | | | 0.6985 | |
| CSE-NV | 40 | 53 |  | 0.9889 | 0.9944 | 0.9791 | | | 0.9938 | |
| CSE-NV | 40 | 75 |  | 0.8772 | 0.9982 | 0.9715 | | | 0.9985 | |
|  |  |  |  |  |  |  | | |  | |
| CSE | 25 | 7 | Isomangiferin | 0.9231 | 0.9265 | 0.9287 | | | 0.9072 | |
| CSE | 25 | 53 |  | 0.8775 | 0.8846 | 0.8905 | | | 0.8772 | |
| CSE | 25 | 75 |  | 0.9060 | 0.9182 | 0.9290 | | | 0.9378 | |
| CSE | 40 | 7 |  | 0.9117 | 0.9189 | 0.9253 | | | 0.9227 | |
| CSE | 40 | 53 |  | 0.8841 | 0.8973 | 0.9095 | | | 0.9444 | |
| CSE | 40 | 75 |  | 0.9577 | 0.9749 | 0.9814 | | | 0.9735 | |
| CSE-NV | 25 | 7 |  | 0.4441 | 0.4470 | 0.4499 | | | 0.2539 | |
| CSE-NV | 25 | 53 |  | 0.6646 | 0.6667 | 0.6687 | | | 0.6606 | |
| CSE-NV | 25 | 75 |  | 0.9660 | 0.9631 | 0.9583 | | | 0.9567 | |
| CSE-NV | 40 | 7 |  | 0.4957 | 0.5004 | 0.5052 | | | 0.6096 | |
| CSE-NV | 40 | 53 |  | 0.9149 | 0.9025 | 0.8886 | | | 0.8862 | |
| CSE-NV | 40 | 75 |  | 0.9864 | 0.9768 | 0.9588 | | | 0.9819 | |

| **TABLE S.4** Continued | | | | | | | | | | |
| --- | --- | --- | --- | --- | --- | --- | --- | --- | --- | --- |
| Sample | Temperature  (° C) | RH (%) | Compound | R^2^_adj_ | | | | | | |
|  |  |  |  | Zero-order | First-order | | Second-order | | Fractional conversion | |
| CSE | 25 | 7 | Vicenin-2 | 0.6346 | 0.6538 | | 0.6727 | | 0.7959 | |
| CSE | 25 | 53 |  | 0.5676 | 0.5892 | | 0.6110 | | 0.8330 | |
| CSE | 25 | 75 |  | 0.4240 | 0.4468 | | 0.4706 | | 0.7928 | |
| CSE | 40 | 7 |  | 0.3501 | 0.3647 | | 0.3795 | | 0.4794 | |
| CSE | 40 | 53 |  | 0.5808 | 0.6050 | | 0.6297 | | 0.8844 | |
| CSE | 40 | 75 |  | 0.9127 | | 0.9060 | | 0.8983 | | 0.8831 |
| CSE-NV | 25 | 7 |  | 0.5766 | | 0.5898 | | 0.6028 | | 0.7223 |
| CSE-NV | 25 | 53 |  | 0.4177 | | 0.4298 | | 0.4420 | | 0.5848 |
| CSE-NV | 25 | 75 |  | 0.3488 | | 0.3696 | | 0.3911 | | 0.6019 |
| CSE-NV | 40 | 7 |  | 0.2746 | | 0.2914 | | 0.3091 | | 0.6634 |
| CSE-NV | 40 | 53 |  | 0.9550 | | 0.9506 | | 0.9447 | | 0.9407 |
| CSE-NV | 40 | 75 |  | 0.8143 | | 0.8058 | | 0.7967 | | 0.7501 |
|  |  |  |  |  | |  | |  | |  |
| CSE | 25 | 7 | Scolymoside | 0.8990 | | 0.8881 | | 0.8762 | | 0.8630 |
| CSE | 25 | 53 |  | 0.7940 | | 0.7940 | | 0.7931 | | 0.7548 |
| CSE | 25 | 75 |  | 0.9162 | | 0.9220 | | 0.9266 | | 0.9073 |
| CSE | 40 | 7 |  | 0.9374 | | 0.9322 | | 0.9258 | | 0.9151 |
| CSE | 40 | 53 |  | 0.8872 | | 0.8874 | | 0.8866 | | 0.8587 |
| CSE | 40 | 75 |  | 0.9756 | | 0.9731 | | 0.9646 | | 0.9704 |
| CSE-NV | 25 | 7 |  | 0.8086 | | 0.8126 | | 0.8163 | | 0.8155 |
| CSE-NV | 25 | 53 |  | 0.5208 | | 0.5219 | | 0.5233 | | 0.3561 |
| CSE-NV | 25 | 75 |  | 0.8868 | | 0.8837 | | 0.8801 | | 0.8485 |
| CSE-NV | 40 | 7 |  | 0.5811 | | 0.5820 | | 0.5829 | | 0.5421 |
| CSE-NV | 40 | 53 |  | 0.7613 | | 0.7593 | | 0.7567 | | 0.7008 |
| CSE-NV | 40 | 75 |  | 0.8678 | | 0.8625 | | 0.8567 | | 0.8226 |
| ^†^Adjusted coefficient of determination; ^‡^*Cyclopia subternata* extract; ^§^*Cyclopia subternata* extract nano-phytosome vesicles; ^¶^Zero order model: C = C_0_$-$Kt; ^††^first-order model : C = C_0_exp($-$Kt); ^‡‡^second-order model: C = C_0_/(1 + C_0_Kt); ^§§^fractional conversion model: C_∞_ + (C_0_-C_∞_)exp($-$Kt) where C is phenolic compound concentration (g/100 g extract), C_0_ is initial phenolic compound concentration (g/100 g extract), t is the time in days and C_∞_ the equilibrium concentration of phenolic compound (g/100 g extract); ^¶¶^3-β-D-glucopyranosyl-4-*O*-β-D-glucopyranosyliriflophenone; ^†††^3′,5′-di-β-D-glucopyranosyl-3-hydroxyphloretin; ^‡‡‡^3′,5′-di-β-D-glucopyranosylphloretin. | | | | | | | | | | |


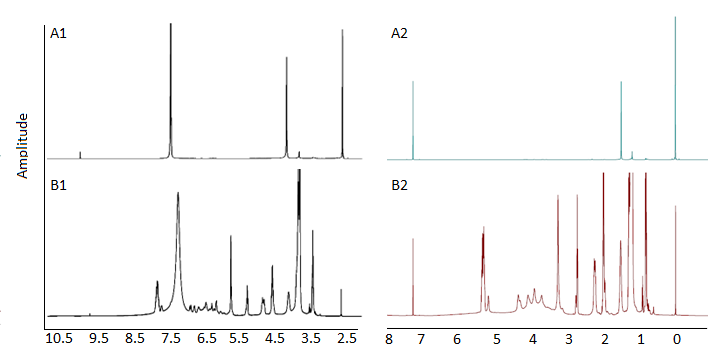


**FIGURE S.1** ^1^H Nuclear magnetic resonance of (A) *Cyclopia subternata* extract (CSE) and (B) lipoid S 40 (fat-free soybean lecithin containing 40% phosphatidylcholine, PC) (1) as obtained and (2) subjected to the selected process conditions for phytosome preparation to confirm that the components are not chemically altered by the process. Differences in intensity and retention times observed for corresponding signals were attributed to different instruments used.

**FIGURE S.2** (1) Brunauer–Emmet–Teller (BET) and (2) Guggenheim–Anderson de Boer (GAB) fittings (indicated by striped lines) and experimental moisture sorption isotherms (indicated by dotted lines) obtained at 25 °C for (A) *Cyclopia subternata* extract (CSE), (B) lipoid S 40 (fat-free soybean lecithin containing 40% phosphatidylcholine, PC) and (C) *Cyclopia subternata* extract nano-phytosome vesicles (CSE-NV).


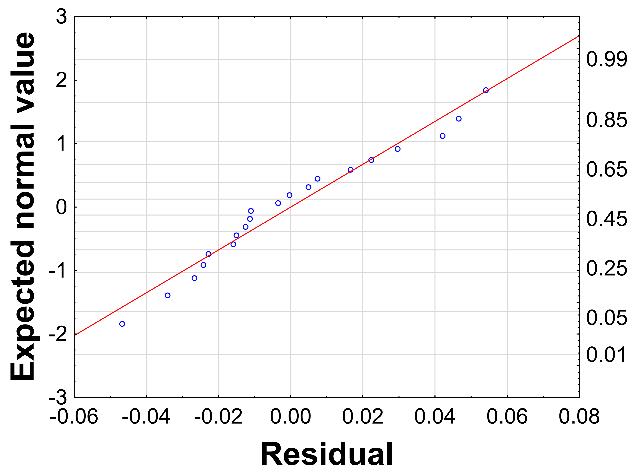

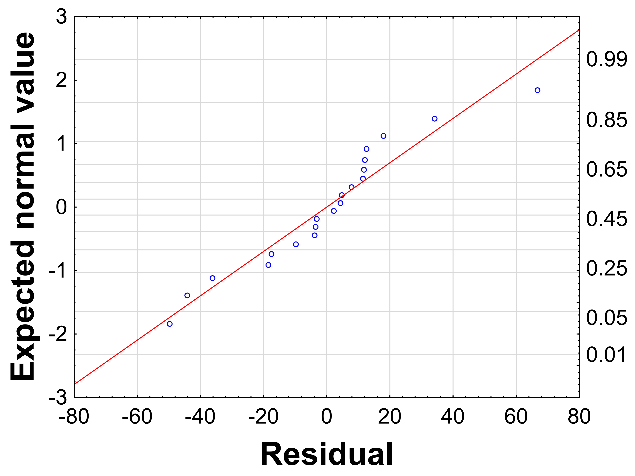

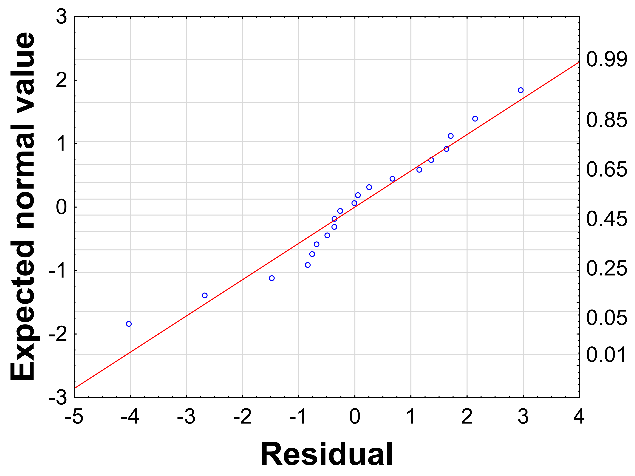

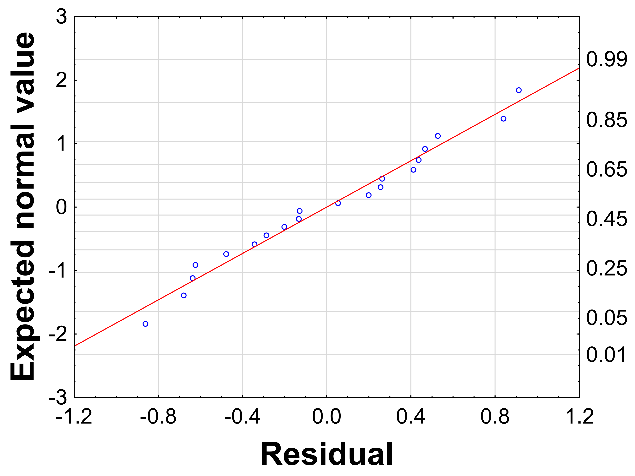

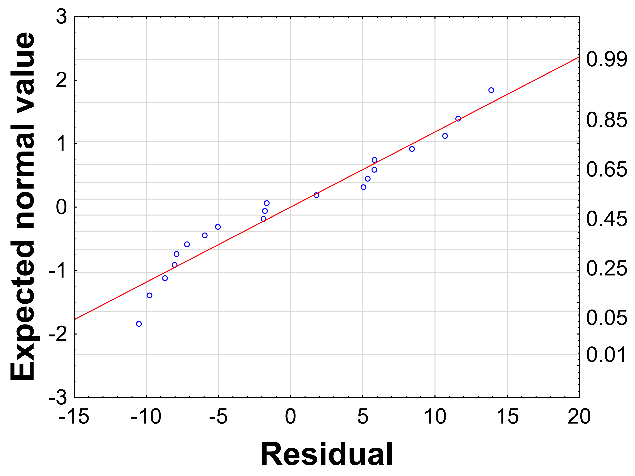

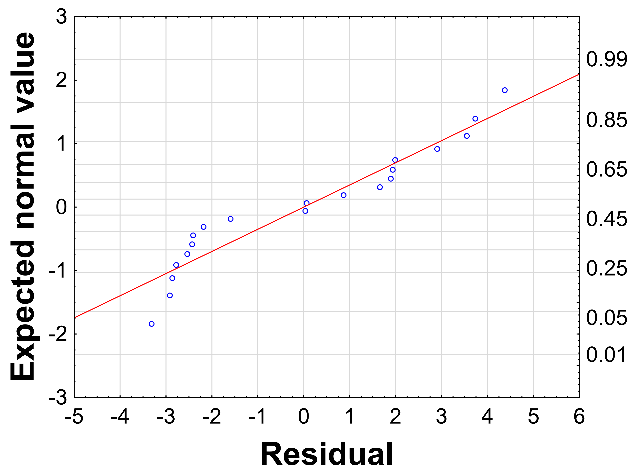


F

E

C

D

A

B

**FIGURE S.3** Residual versus the expected normal value plots for responses of a central composite design to produce *Cyclopia subternata* extract nano-phytosome vesicles (CSE-NV). (A) particle size, (B) polydispersity index, (C) zeta potential, (D) yield, (E) encapsulation efficiency, (F) loading capacity.

**FIGURE S.4** Second-order kinetic fitting (indicated by (f)) and experimental data of (A) 3-β-D-glucopyranosyl-4-*O*-β-D-glucopyranosyliriflophenone (IDG); (B) 3′,5′-di-β-D-glucopyranosyl-3-hydroxyphloretin (HPDG); (C) 3′,5′-di-β-D-glucopyranosylphloretin (PDG) and (D) eriocitrin content in *Cyclopia subternata* extract (CSE) and CSE nano-phytosome vesicles (CSE-NV) during storage at different temperature and relative humidity (RH) combinations (7, 53 and 75% RH at (1) 25 °C and (2) 40 °C).

**FIGURE S.5** Second-order kinetic fitting (indicated by (f)) and experimental data of (A) hesperidin; (B) mangiferin; (C) isomangiferin and (D) vicenin-2 content in *Cyclopia subternata* extract (CSE) and CSE nano-phytosome vesicles (CSE-NV) during storage at different temperature and relative humidity (RH) combinations (7, 53 and 75% RH at (1) 25 °C and (2) 40 °C).

**FIGURE S.6** Second order kinetic fitting (indicated by (f)) and experimental data of (A) scolymoside content in *Cyclopia subternata* extract (CSE) and CSE nano-phytosome vesicles (CSE-NV) during storage at different temperature and relative humidity (RH) combinations (7, 53 and 75% RH at (1) 25 °C and (2) 40 °C
